# *Borrelia burgdorferi* infection induces long-term memory-like responses in macrophages with tissue-wide consequences in the heart

**DOI:** 10.1371/journal.pbio.3001062

**Published:** 2021-01-04

**Authors:** Diego Barriales, Itziar Martín-Ruiz, Ana Carreras-González, Marta Montesinos-Robledo, Mikel Azkargorta, Ibon Iloro, Iraide Escobés, Teresa Martín-Mateos, Estibaliz Atondo, Ainhoa Palacios, Monika Gonzalez-Lopez, Laura Bárcena, Ana R. Cortázar, Diana Cabrera, Ainize Peña-Cearra, Sebastiaan M. van Liempd, Juan M. Falcón-Pérez, Miguel A. Pascual-Itoiz, Juana María Flores, Leticia Abecia, Aize Pellon, Maria Luz Martínez-Chantar, Ana M. Aransay, Alberto Pascual, Felix Elortza, Edurne Berra, José Luis Lavín, Héctor Rodríguez, Juan Anguita

**Affiliations:** 1 Inflammation and Macrophage Plasticity Laboratory, CIC bioGUNE-BRTA (Basque Research and Technology Alliance), Derio, Spain; 2 Proteomics Platform, ProteoRed-ISCIII, CIC bioGUNE-BRTA, Derio, Spain; 3 Physiopathology of the Hypoxia-Signaling Pathway Laboratory, CIC bioGUNE-BRTA, Derio, Spain; 4 Genomic Analysis Platform, CIC bioGUNE-BRTA, Derio, Spain; 5 Metabolomics Platform, CIC bioGUNE-BRTA, Derio, Spain; 6 Ikerbasque, Basque Foundation for Science, Bilbao, Spain; 7 Department of Animal Medicine and Surgery, Veterinary Faculty, Universidad Complutense de Madrid, Madrid, Spain; 8 Liver Diseases Laboratory, CIC bioGUNE-BRTA, Derio, Spain; 9 CIBERehd, Instituto de Salud Carlos III, Madrid, Spain; 10 Instituto de Biomedicina de Sevilla, Hospital Universitario Virgen del Rocío/CSIC/Universidad de Sevilla, Seville, Spain; 11 Bioinformatics Service, CIC bioGUNE-BRTA, Derio, Spain; Washington University in St. Louis, UNITED STATES

## Abstract

Lyme carditis is an extracutaneous manifestation of Lyme disease characterized by episodes of atrioventricular block of varying degrees and additional, less reported cardiomyopathies. The molecular changes associated with the response to *Borrelia burgdorferi* over the course of infection are poorly understood. Here, we identify broad transcriptomic and proteomic changes in the heart during infection that reveal a profound down-regulation of mitochondrial components. We also describe the long-term functional modulation of macrophages exposed to live bacteria, characterized by an augmented glycolytic output, increased spirochetal binding and internalization, and reduced inflammatory responses. In vitro, glycolysis inhibition reduces the production of tumor necrosis factor (TNF) by memory macrophages, whereas in vivo, it produces the reversion of the memory phenotype, the recovery of tissue mitochondrial components, and decreased inflammation and spirochetal burdens. These results show that *B*. *burgdorferi* induces long-term, memory-like responses in macrophages with tissue-wide consequences that are amenable to be manipulated in vivo.

## Abstract 2

Lyme carditis is a manifestation of Lyme disease characterized by episodes of atrioventricular block and additional cardiomyopathies. This study describes the proteomic and transcriptomic changes in the heart upon infection with Borrelia burgdorferi, and identifies innate immune memory hallmarks specific to the response to the spirochete that are amenable to therapeutic manipulation.

## Introduction

Lyme borreliosis, caused by the infection with the spirochete, *Borrelia burgdorferi*, is the most common arthropod-borne infection in the Northern Hemisphere with around 300,000 cases every year in the United States [1,2] and approximately 250,000 new cases yearly in Europe [3]. Symptoms associated with infection may be debilitating and long-lasting despite appropriate antibiotic treatment. The initial deposition of the bacteria in the skin is followed by the intradermal and hematogenous dissemination of the spirochete, which results in the invasion of different tissues and organs, including the joints and the nervous and cardiovascular systems. *B*. *burgdorferi* shows a marked tropism for cardiac tissue [4] where it can persist for months to years even after antibiotic treatment [5–7] and in spite of the development of strong immune responses. Lyme carditis represents 0.3% to 4% of Lyme borreliosis cases in Europe [8] and 4% to 10% in the US [9]. Moreover, 80% to 90% of Lyme carditis patients suffer atrioventricular electrical block of varying degrees [10], while myocarditis, myocardial infarction, coronary aneurisms, pericarditis, pancarditis, dilated cardiomyopathy, or endocarditis have been reported in a smaller percentage of infected individuals [11]. Although apparent in a small percentage of Lyme borreliosis cases, 11 fatalities associated with Lyme carditis have been reported since 1985 (https://www.cdc.gov/lyme/signs_symptoms/lymecarditis.html) [4].

The heart is the most energy-consuming organ in the human body [12]. More than 40% of the cytoplasmic space in adult cardiomyocytes and 50% of the volume of the myofibrils is occupied by mitochondria [13,14]. This high-capacity mitochondrial system is capable of oxidizing fuels such as fatty acids, which are the predominant energy substrate for the adult heart [15]. Abnormalities in the mitochondrial function cause several pathologies [16–18]. During sepsis, both a deficit in energy production and alterations in the source of energy substrates are associated with impaired cardiac function [19], in part by the myocardial infiltration of immune cells [20], accompanied by a strong activation of the cytokine system [21].

The existence of long-term consequences of the stimulation of macrophages with certain simple (e.g., beta glucans) or complex (e.g., Bacille Calmette–Guérin (BCG), the mycobacterial vaccine strain) stimuli has been termed “innate immune memory” [22,23]. This concept originally evolved from observations in BCG-vaccinated individuals in which a level of protection against disparate pathogens was identified [24]. Innate immune memory has been defined in terms of the induction of soluble factors (i.e., pro-inflammatory cytokines) [25]. Responses identified as memory have been divided into innate immune training and tolerance, the difference being the nature of the secondary response (heightened versus reduced). Innate immune memory is replicated in vitro by the use of a primary stimulus, a period of resting, and a different secondary stimulus. Although the mechanisms underlying the development of innate immune memory are not completely known, both variations in metabolism (Warburg effect) mediated by the AKT/mTOR/HIF axis and epigenetic changes are known to occur [24,26,27]. The impact of this previous experience on the ability of monocytes/macrophages to internalize/phagocytose microorganisms has been, however, largely unaddressed, in spite of the relevance of this process in the elimination of pathogens and the intimate relationship between phagocytosis and the inflammatory output of macrophages [28,29]. Moreover, the response to live and killed microorganisms is vastly different, both quantitatively and qualitatively [30–32]. Therefore, the phenotypic and regulatory mechanisms of innate immune memory cells against pathogens that are able to establish persistent infections are lacking.

We have recently identified transcriptional traits and signaling pathways associated with the short-time stimulation of monocytes/macrophages from both human and murine origin with *B*. *burgdorferi*, such as peroxisome proliferator-activated receptor (PPAR) and the Toll-like receptor (TLR) family member, CD180 [33]. However, macrophages are likely exposed to *B*. *burgdorferi* during prolonged periods of time. Therefore, the response of these cells may be differentially modulated over time due to transcriptional, epigenetic, or metabolic changes, including the integration of primary (to the spirochete) and secondary (to metabolites or inflammatory/anti-inflammatory) factors. Here, we have analyzed the long-term responses of macrophages to the spirochete with an emphasis on the ability to control bacterial phagocytosis and the ensuing pro-inflammatory response, as well as the regulatory control of these responses. We also show that the spatially circumscribed interaction between *B*. *burgdorferi* and macrophages has tissue-wide transcriptional and functional consequences that are directly correlated with the inflammatory output in the heart.

## Results

### Infection with *B*. *burgdorferi* induces whole tissue changes in the heart

**Fig 1:**
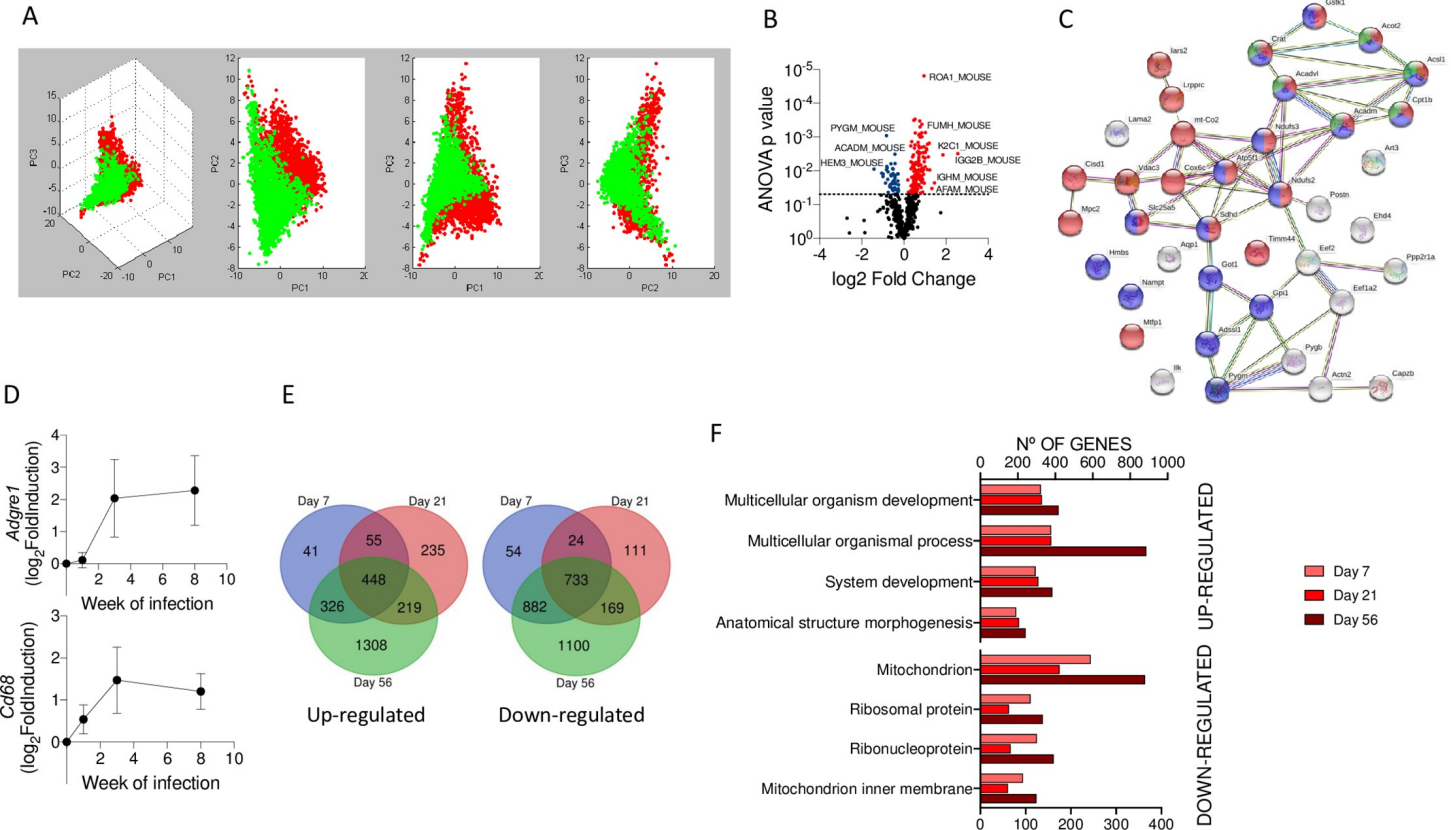
Infection with *B*. *burgdorferi* results in broad transcriptional and proteomic changes in the heart. (A) Tridimensional PCA distinguishing between infected (green dots) and uninfected (red dots) auricular areas. The data were obtained from 3 infected and 3 uninfected mouse hearts. (B) Volcano plot representing the number of proteins differentially represented in the hearts of infected mice compared to uninfected hearts. (C) Protein network analysis of proteins underrepresented in infected hearts compared to uninfected controls. Red dots represent mitochondrial proteins. Blue dots represent proteins associated with fatty acid metabolism. Green dots represent proteins associated with metabolism, according to GO-BP. (D) Venn diagrams showing the number of genes up-regulated (left panel) or down-regulated (right panel) in 7-, 21-, and 56-day-infected mouse hearts compared to uninfected controls. The data were generated by the transcriptional analysis of 6–10 mice per group. (E) Relative expression levels (expressed as log_2_ fold induction) of the *Adgre1* (top panel) and *Cd68* (bottom panel) genes at days 7 (week 1), 21 (week 3), and 56 (week 8) postinfection compared to uninfected controls (value = 0). Groups of 3–6 mice were used in each time point. (F) Number of genes up- (top) and down-regulated (bottom) during the time course of infection involved in different biological processes obtained from the GO BP database. The data underlying the graphs in Fig 1 can be found in S1 Supporting Information, S1 Data, PXD019605, and GSE152168. ANOVA, analysis of variance; GO-BP, Gene Ontology Biological Process; PCA, principal component analysis.

We first analyzed by tissue MALDI Imaging (MALDI-IMS) global proteomic changes in the hearts of 3-week-infected mice with *B*. *burgdorferi* compared to uninfected controls. The analysis of the tissues was performed with resolution close to 100 μm and could clearly distinguish ventricular and auricular areas, both spectrally and through principal component analysis (PCA) (S1A and S1B Fig). We then analyzed the auricular regions of infected and control hearts in search of molecular markers associated with infection. PCA showed differences that depended on the infectious status of the hearts (Fig 1A). We detected several peaks with receiving operating characteristic (ROC) curves above the threshold of 0.8. For example, a band of 5,311 Da appeared intense in the healthy tissue, while it almost disappeared in the infected hearts (S1C Fig). In contrast, the band of 7,017 Da was of an increased intensity in the infected tissue (S1C Fig). Those corresponding to 11.3, 13.8, and 14 kDa were also of higher intensity in the infected hearts, while showing a similar localization pattern.

In order to identify specific proteins affected by the infectious status of the hearts, we analyzed by label-free mass spectrometry extracts obtained from the basal region of the tissue. We identified 596 proteins represented by at least 2 peptides, of which 198 were differentially expressed (analysis of variance (ANOVA), *p* < 0.05, absolute fold induction >1.25; 155 up-regulated, 41 down-regulated) in infected tissue (Fig 1B). Proteins overexpressed in the infected hearts contained an overrepresentation of elements involved in cardiac muscle contraction and contractile fiber part (S1 Table). Strikingly, 23 of the 41 down-regulated proteins were mitochondrial components (Fig 1C) and coincided with elements involved in metabolic pathways, including fatty acid metabolism (Fig 1C). On the other hand, 13 of 155 up-regulated proteins (ATP5B, ATP5H, ATP5J, DLD, ETFB, FH1, GLO1, NDUFA5, NDUFAB1, NDUFB9, NDUFS5, and NDUFV2) corresponded to tricarboxylic acid cycle (TCA) and respiratory chain proteins (S1 Table). Of these, several belong to the NADH dehydrogenase complex and ATP synthase. These data suggested a compensatory mechanism for the increased generation of ATP.

To further analyze the cardiac effects resulting from *B*. *burgdorferi* infection, we analyzed the transcriptional profile of hearts from mice that had been infected for 7, 21, and 56 days, representing early, acute, and resolving infection [34]. The analysis of *Adgre1* (encoding the surface protein, F4/80) and *Cd68* expression allowed us to monitor macrophage infiltration in the cardiac tissue (Fig 1D). The infiltration of macrophages was minimal at day 7 of infection, augmented significantly at day 21, and remained at similar levels throughout day 56 (Fig 1D). A total of 2,632 genes were increased at any time point of infection, with 448 genes up-regulated at all times analyzed (Fig 1E). Similarly, 3,073 genes were found to be down-regulated at any time point of infection, with 733 down-regulated at all time points (Fig 1E). Similar Gene Ontology Biological Processes (GO-BP) were altered regardless of infection time, when up-regulated genes were analyzed (Fig 1F, top). These functions included multicellular organism development and process, system development, and anatomical structure morphogenesis (Fig 1F, top). Strikingly, a sizeable proportion of down-regulated genes corresponded to mitochondrial, and to a lesser extent, ribosomal proteins (Fig 1F, bottom).

We then compared the proteomic and transcriptomic data. We matched the proteins and their corresponding genes and assessed their regulation status at 21 days of infection. Of the genes down-regulated in infected hearts, several corresponded to proteins that showed up-regulation (S2A Fig). On the other hand, we found that the proteins down-regulated correlated with genes that either did not change expression or were down-regulated in the infected tissue (S2B Fig). However, several of the up-regulated proteins showed genes with lower expression in the infected hearts (S2B Fig). Overall, these data suggested that several of the proteins maintained a high level of expression in spite of the down-regulation of the expressing gene, possibly due to posttranslational and stabilizing modifications.

Overall, these data show that the infection of the mammalian host with *B*. *burgdorferi* results in tissue-wide gene and protein expression changes, which includes the down-regulation of mitochondrial components.

### *B*. *burgdorferi* induces a differential transcriptional profile in acutely stimulated and memory macrophages

Cardiac inflammation induced by *B*. *burgdorferi* is dependent on the interaction of the spirochete with macrophages and other cell types, such as invariant NKT (iNKT) or T cells [35]. The relevance of transcriptional and biochemical reprogramming events in macrophages has been recently highlighted [36]. However, the functional implications of these changes in the macrophage response have not been fully addressed, especially in the context of persistent infections where macrophages are reexposed to the pathogen over time. In order to understand the functional consequences of the persistent activation of macrophages with *B*. *burgdorferi* compared to their acute stimulation, we stimulated murine bone marrow-derived macrophages (BMMs) for 48 hours, washed them, and restimulated them with the spirochete for 16 to 20 hours (memory macrophages, condition BB; S3A Fig). Acutely stimulated macrophages were processed in parallel, except with no stimulation the first 48 hours (condition UB). Unstimulated (condition UU) and stimulated and rested (condition BU) macrophages were also analyzed (S3A Fig).

**Fig 2:**
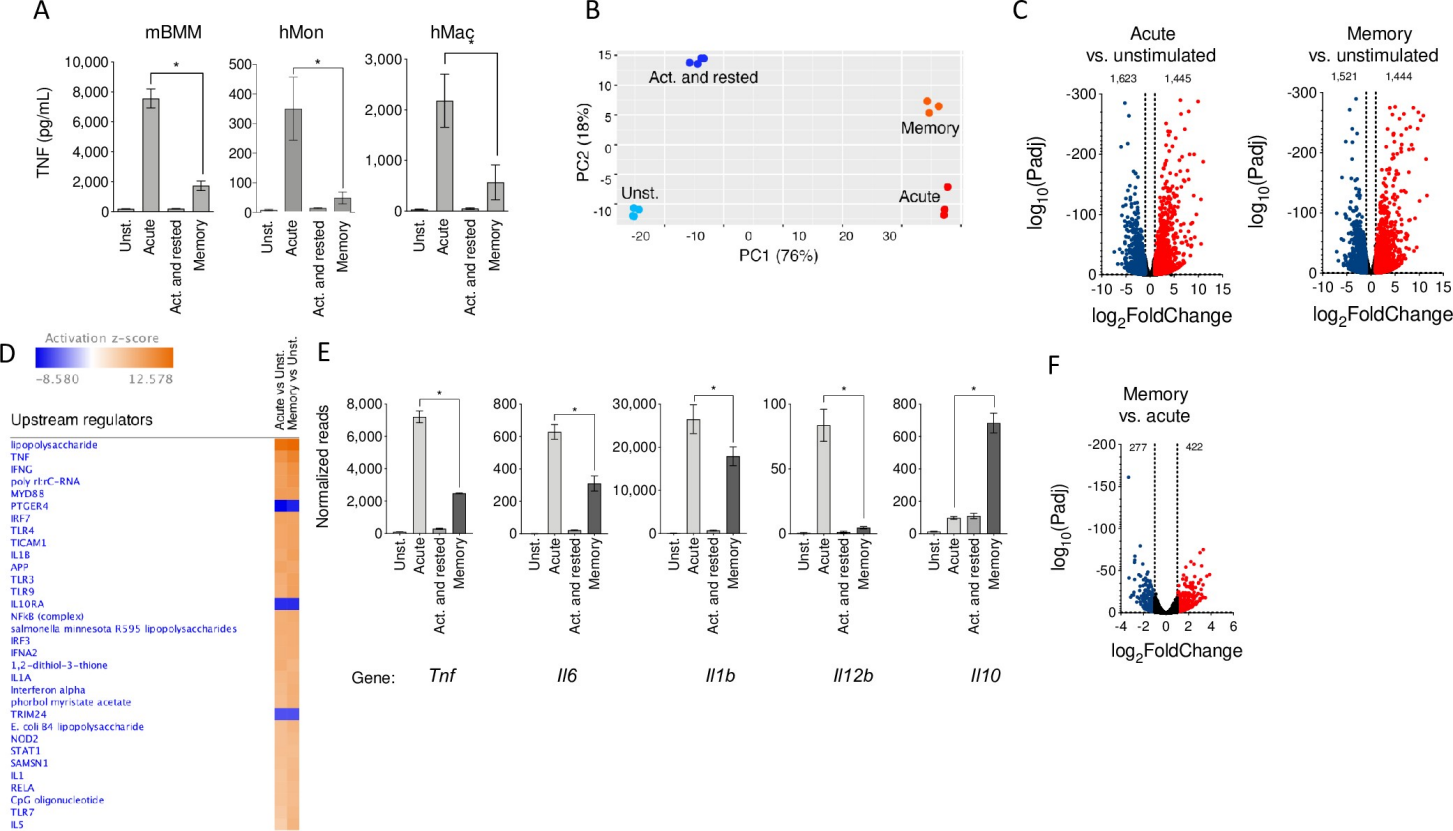
*B*. *burgdorferi* induces long-term responses in macrophages that modulate the transcriptome and pro-inflammatory cytokine production. (A) TNF production by mBMM, hMons, and hMacs acutely and restimulated with *B*. *burgdorferi*, compared to unstimulated and activated and rested cells. The figure represents the results from 10 independent mice and hMons from 5 individuals. *B*. *burgdorferi* were used at an MOI of 25 (B) PCA of the transcriptome of unstimulated, acutely stimulated, activated and rested, and restimulated macrophages. (C) Volcano plots representing genes differentially expressed by acutely stimulated (left panel) and restimulated (right panel) macrophages vs. unstimulated cells. (D) Upstream pathways regulated in acute and memory macrophages compared to unstimulated BMMs. Processes that showed activation are indicated in orange, whereas those that were repressed are presented in blue. The color intensities are representative of the calculated Z value for each process by IPA. (E) mRNA expression levels of pro- and anti-inflammatory cytokines in BMMs stimulated with the spirochete. The normalized reads for each condition are represented. *, *p* < 0.05. (F) Volcano plot showing the DE of genes when comparing acute and memory macrophages. The red dots represent genes up-regulated in memory macrophages (422), and the blue dots indicate genes that are down-regulated (277). The DE of genes was set as an absolute value log2 fold induction of 1 and Padj < 0.05. The data underlying the graphs in Fig 2 can be found in S2 Data and GSE125503. BMM, bone marrow-derived macrophage; DE, differential expression; hMac, human monocyte-derived macrophage; hMon, human peripheral monocyte; IPA, Ingenuity Pathway Analysis; mBMM, murine bone marrow-derived macrophage; MOI, multiplicity of infection; mRNA, messenger RNA; PCA, principal component analysis; TNF, tumor necrosis factor.

We then compared the acutely stimulated and memory macrophages for their capacity to produce pro-inflammatory cytokines and their phagocytic activity when stimulated with *B*. *burgdorferi*. Memory macrophages produced decreased tumor necrosis factor (TNF) levels in response to the bacterium, compared to acutely stimulated cells (Fig 2A). The analysis under the same conditions of purified CD14^+^ cells and monocyte-derived macrophages from peripheral blood of healthy donors confirmed that in human monocytes, the previous exposure to live *B*. *burgdorferi* (condition BB) renders the cells hyporesponsive to the spirochete in a subsequent encounter compared to acutely stimulated monocytes (condition UB) (Fig 2A).

To identify transcriptional traits specifically induced in naive and previously activated macrophages with *B*. *burgdorferi*, we analyzed the transcription profiles of BMMs by RNA sequencing (RNA-seq). We analyzed BMMs that had been stimulated with the spirochete under the 4 conditions mentioned above. Each of the conditions showed distinct transcriptional profiles, as seen in PCA (Fig 2B) and sample distance matrix analysis (S3B Fig). In spite of the similarities between the unstimulated and the stimulated and rested conditions (S3B Fig), 1,334 genes showed differential regulation when using cutoff values of 1 for the absolute log_2_ fold change and Padj < 0.05 (693 up and 641 down; S3C Fig).

On the other hand, the comparison to unstimulated macrophages with acutely stimulated and memory cells revealed similar number of up-regulated and down-regulated genes (Fig 2C). Indeed, among the genes differentially expressed under the acute stimulation and memory conditions, a majority (2,154) were found to be common (S3D Fig). Ingenuity Pathway Analysis (IPA) showed that the genes regulated in memory macrophages were consistent with pathways activated by the acute stimulation of BMMs with *B*. *burgdorferi* [33], including interferons, TLRs, nucleotide-binding oligomerization domain-like receptors (NLRs), and cytokines such as interleukin (IL)-1β (Fig 2D). The transcriptional analysis of pro-inflammatory cytokine production (*Tnf*, *Il6*, *Il1b*, and *Il12b*) confirmed the pattern observed for TNF by ELISA, while the levels of *Il10* transcripts were highly up-regulated in memory cells (Fig 2E). However, as observed in acutely stimulated cells [33], the IL-10R-dependent signaling pathway was also significantly repressed in memory macrophages, compared to unstimulated controls (Fig 2D). Despite these similarities, a sizeable number of genes appeared differentially regulated in acutely stimulated and memory macrophages (S3E Fig). In fact, the comparison of both conditions showed that 422 genes were up-regulated in memory macrophages, while 277 were down-regulated compared to the acute stimulation of the cells (Fig 2F).

In order to assess whether the memory phenotype had been acquired after the first stimulation, we also compared the transcriptional profile of activated and rested (condition BU) and memory macrophages (condition BB). Using Generally Applicable Gene-set Enrichment (GAGE) for pathway analysis [37], we observed that the only pathways significantly down-regulated in BU macrophages were those corresponding to the recognition of infectious agents (S2 Table), with no differentially up-regulated pathways. These data indicated that the difference between the BU and the BB conditions arise due to the response to the pathogen in the second stimulation. The data also suggest that the memory phenotype is acquired during the first stimulation period. Indeed, the analysis by flow cytometry of green fluorescent protein (GFP)-labeled *B*. *burgdorferi* after 48 hours of incubation indicated the lack of this fluorescent marker (S4 Fig), suggesting that the spirochetes had been degraded at this time point.

### Previous exposure to *B*. *burgdorferi* increases the ability of macrophages to bind the spirochete

**Fig 3:**
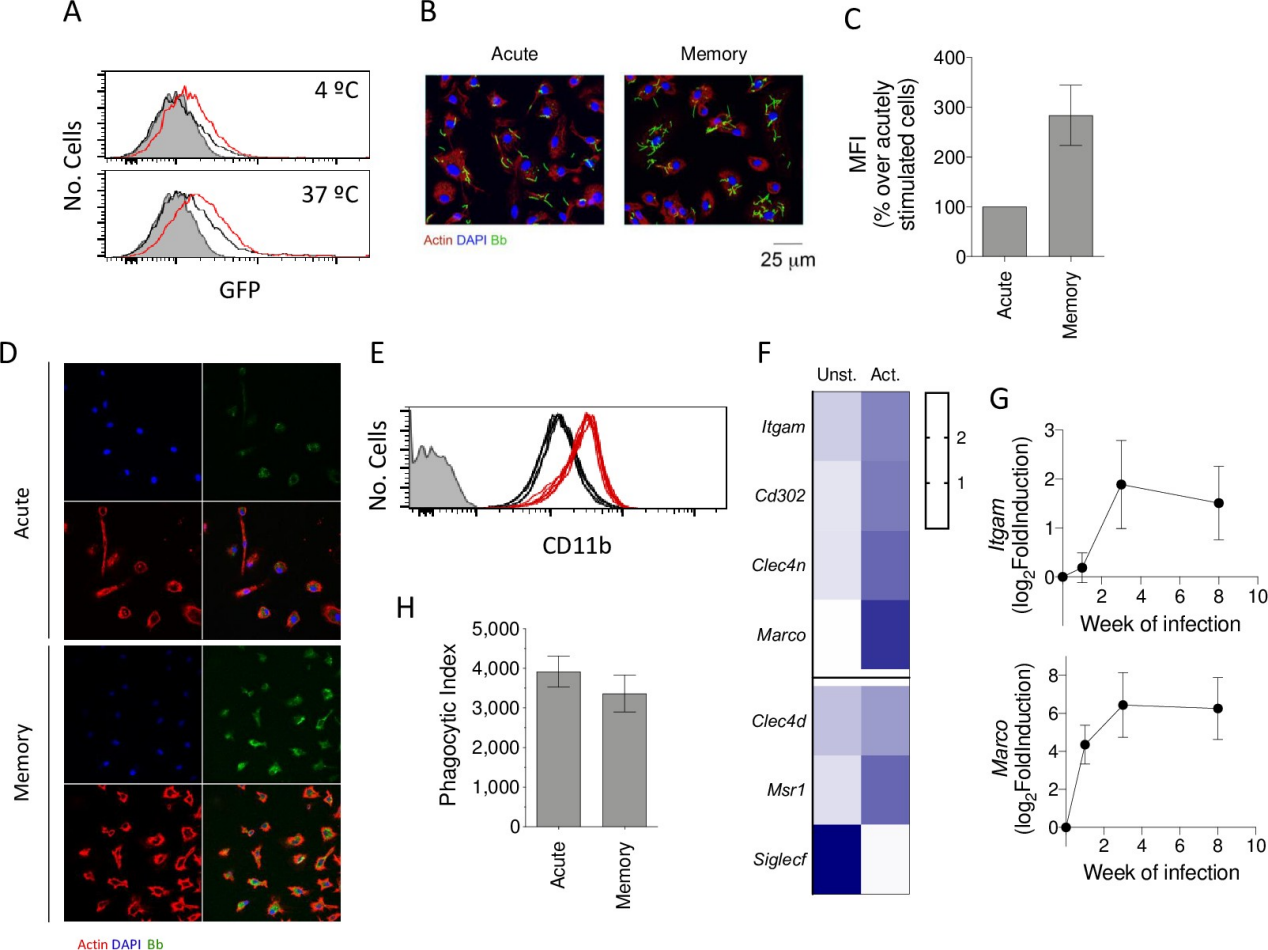
Previously exposed murine macrophages show increased binding and internalization of *B*. *burgdorferi*. (A) *B*. *burgdorferi* binding (upper panel) and internalization (lower panel) by naïve (black histograms) and memory (red histograms) BMMs. The gray histograms represent BMMs with no spirochetes added. (B) Confocal image showing binding of GFP-*B*. *burgdorferi* to acute and memory macrophages. The cells were incubated at 4°C for 2 hours, fixed, and stained with phalloidin and DAPI. (C) Increased MFI of memory macrophages over acute cells, incubated for 2 hours at 4°C. The data are presented as percentage increase over acutely stimulated macrophages. (D) Confocal images showing the internalization of GFP-*B*. *burgdorferi* by acute and memory hMacs. The cells were incubated at 37°C for 2 hours, fixed, and stained with phalloidin and DAPI. (E) Expression of CD11b by unexposed (black histograms) and previously experienced (red histograms) macrophages. The figure represents the results of 6 independent mice. (F) Heat map representing expression levels of genes encoding for macrophage receptors comparing unstimulated and *B*. *burgdorferi*–exposed BMMs. The blue color intensity (right) represents the relative level of expression. (G) Log_2_ fold induction of the *Itgam* (encoding CD11b) and *Marco* genes in the hearts of infected mice, determined at 0, 7 (1 week), 21 (3 weeks), and 56 (8 weeks) days of infection. Groups of 3–6 mice were used for each time point. (H) Phagocytic index of acute and memory macrophages, as determined by the formula: % GFP cells (test) × MFI (test) − % GFP cells (4°C control) × MFI (4°C control). This figure represents the results from 6 independent experiments including groups of 3–5 mice per experiment. The data underlying the graphs in Fig 3 can be found in S3 Data, GSE125503, and GSE152168. BMM, bone marrow-derived macrophage; GFP, green fluorescent protein; hMac, human monocyte-derived macrophage; MFI, mean fluorescence intensity.

The inflammatory response of macrophages to *B*. *burgdorferi* is intimately associated with their phagocytic capacity [29,30,38]. We therefore analyzed the capacity of previously activated macrophages to both bind and internalize the spirochete compared to unstimulated cells. The analysis by flow cytometry revealed an augmented capacity to bind the spirochete of macrophages previously activated with *B*. *burgdorferi* when incubated at 4°C (Fig 3A–3C). This resulted in increased internalization when the cells were further incubated at 37°C (Fig 3A) as observed also in human monocyte-derived macrophages by confocal microscopy (Fig 3D). We then analyzed the expression levels of phagocytic receptors under both conditions in our RNA-seq data. The surface expression levels of CD11b [39–41] were higher in previously stimulated cells, compared to unexposed controls (Fig 3E), which could be observed at the gene expression level (*Itgam*, Fig 3F). We also found increased expression levels of genes involved in the binding of *B*. *burgdorferi* to macrophages [42], including *Marco*, *Cd302* (encoding the receptor CLEC4B1), *Clec4n*, *Clec4d*, and *Msr1*, while *Siglecf* (encoding the receptor Siglec5) was highly down-regulated (Fig 3F). The analysis of *Itgam* and *Marco* expression in vivo confirmed the increased expression of these receptors in the hearts of the infected mice, although with different kinetics (Fig 3G). These data suggest that the enhanced binding capacity of memory macrophages is due to the increased expression of phagocytic receptors.

In order to analyze the internalization rate of the spirochete independently of the binding capacity of the cells, we compared the phagocytic index [42] of macrophages previously stimulated with the spirochete and unstimulated cells. This analysis revealed similar internalization rates for *B*. *burgdorferi* (Fig 3H), indicating that the higher internalization observed in macrophages previously activated was due to the increased ability of these cells to bind the bacterium rather than an augmented capacity to internalize the spirochete once bound to the cell.

Altogether, these data show that macrophages exposed to *B*. *burgdorferi* augment the capacity to bind and subsequently internalize the spirochete, albeit with the production of reduced levels of TNF.

### *B*. *burgdorferi*–induced *Irf4* expression modulates pro-inflammatory cytokine production in memory macrophages

**Fig 4:**
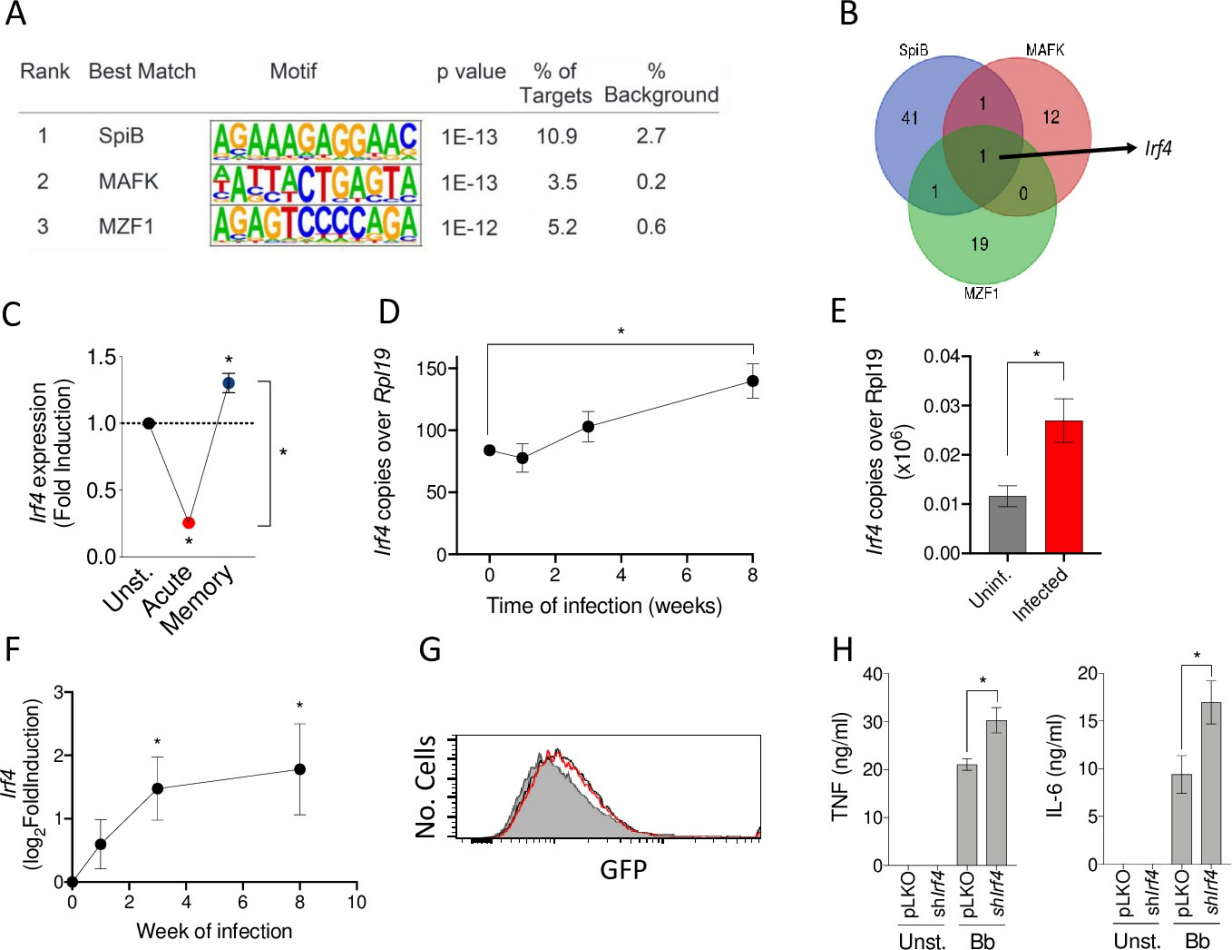
*B*. *burgdorferi*–induced *Irf4* expression modulates the induction of pro-inflammatory cytokines in previously experienced macrophages. (A) Motifs associated with the expression of genes specifically up-regulated in memory macrophages compared to acutely stimulated cells. (B) Venn diagram showing the genes that are co- or differentially regulated by the transcription factors, SpiB, MAFK, and MZF1. The only gene regulated by the 3 transcription factors is *Irf4*. (C) *Irf4* gene expression in acute, memory, and non-stimulated macrophages determined by real-time PCR relative to *Rpl19*. The results represent 4 independent mice. *, Student *t* test, *p* < 0.05. (D) Expression levels of *Irf4* in peripheral blood of infected mice over time, as determined by real-time PCR relative to *Rpl19*. Groups of 3–6 mice were employed for each time point. *, Student *t* test, *p* < 0.05. (E) *Irf4* expression of 21-day-infected hearts vs. uninfected controls by real-time PCR relative to *Rpl19*. (F) Log_2_ fold induction of *Irf4* in infected hearts at different time points of infection. Groups of 3–6 mice were employed for each time point. *, Student *t* test, *p* < 0.05. (G) Phagocytosis by BMMs infected with lentivirus containing sh*Irf4* (red histogram) or the control vector, pLKO (black histogram). The gray histogram represents a 4°C control. The cells were coinfected with lentivirus containing 2 different shRNA sequences (TRCN0000081548; TRCN0000081549). The average level of silencing of the *Irf4* was calculated to be at 23 ± 0.05%. (H) TNF and IL-6 induction by *B*. *burgdorferi* in *Irf4*-silenced and control BMMs. The cells were stimulated for 20 hours, followed by the measurement of the cytokines in the supernatants by ELISA. The data underlying the graphs in Fig 4 can be found in S4 Data, GSE125503, and GSE152168. BMM, bone marrow-derived macrophage; GFP, green fluorescent protein; IL, interleukin; PCR, polymerase chain reaction; shRNA, short hairpin RNA; TNF, tumor necrosis factor.

We then performed a comparative transcription factor enrichment analysis of acutely stimulated and memory macrophages using the HOMER package with our RNA-seq data [43]. We identified a set of 3 transcription factors putatively responsible for the transcriptional changes in memory macrophages compared to acutely stimulated cells: SpiB, MAFK, and MZF1 (Fig 4A). The analysis of the genes with changed expression in memory macrophages, compared to acutely stimulated cells (S3 Table), identified *Irf4* as the only gene regulated by all 3 transcription factors (Fig 4B). *Irf4* expression was significantly reduced upon the acute exposure of macrophages to the spirochete when compared to unstimulated cells, while memory macrophages showed increased expression levels when compared to both unstimulated and acutely stimulated cells (Fig 4C). The analysis over time of *Irf4* expression in peripheral blood of *B*. *burgdorferi*–infected mice showed a slight reduction at the initial phases (week 1 of infection) that increased over time, being significantly higher than in noninfected controls at week 8 of infection (Fig 4D). We also tested by real-time polymerase chain reaction (PCR) the level of expression of *Irf4* in 3-week-infected hearts. In this organ, the expression of the gene was significantly higher than in noninfected controls (Fig 4E). Indeed, the analysis of the transcriptomics of the heart over the time of infection showed increased expression of *Irf4* a soon as day 7 of infection, being significantly higher as the disease progressed (Fig 4F).

We therefore assessed the role of IRF4 on the response of phagocytic cells to the spirochete. First, we generated RAW 264.7 cells with stable expression of silencing RNA for *Irf4* (sh*Irf4*). Remarkably, *Irf4* silencing did not affect the internalization of *B*. *burgdorferi* (S5A Fig) but resulted in increased TNF production (S5B Fig). Similarly, *Irf4* silencing in BMMs did not affect phagocytosis of *B*. *burgdorferi* (Fig 4G) but resulted in significantly increased levels of both TNF and IL-6 (Fig 4H). These data show that phagocytosis and the inflammatory output of macrophages can be independently regulated. They also demonstrate that IRF4 modulates the inflammatory output of macrophages in response to *B*. *burgdorferi*.

### Long-term stimulation with *B*. *burgdorferi* induces metabolic changes in macrophages

**Fig 5:**
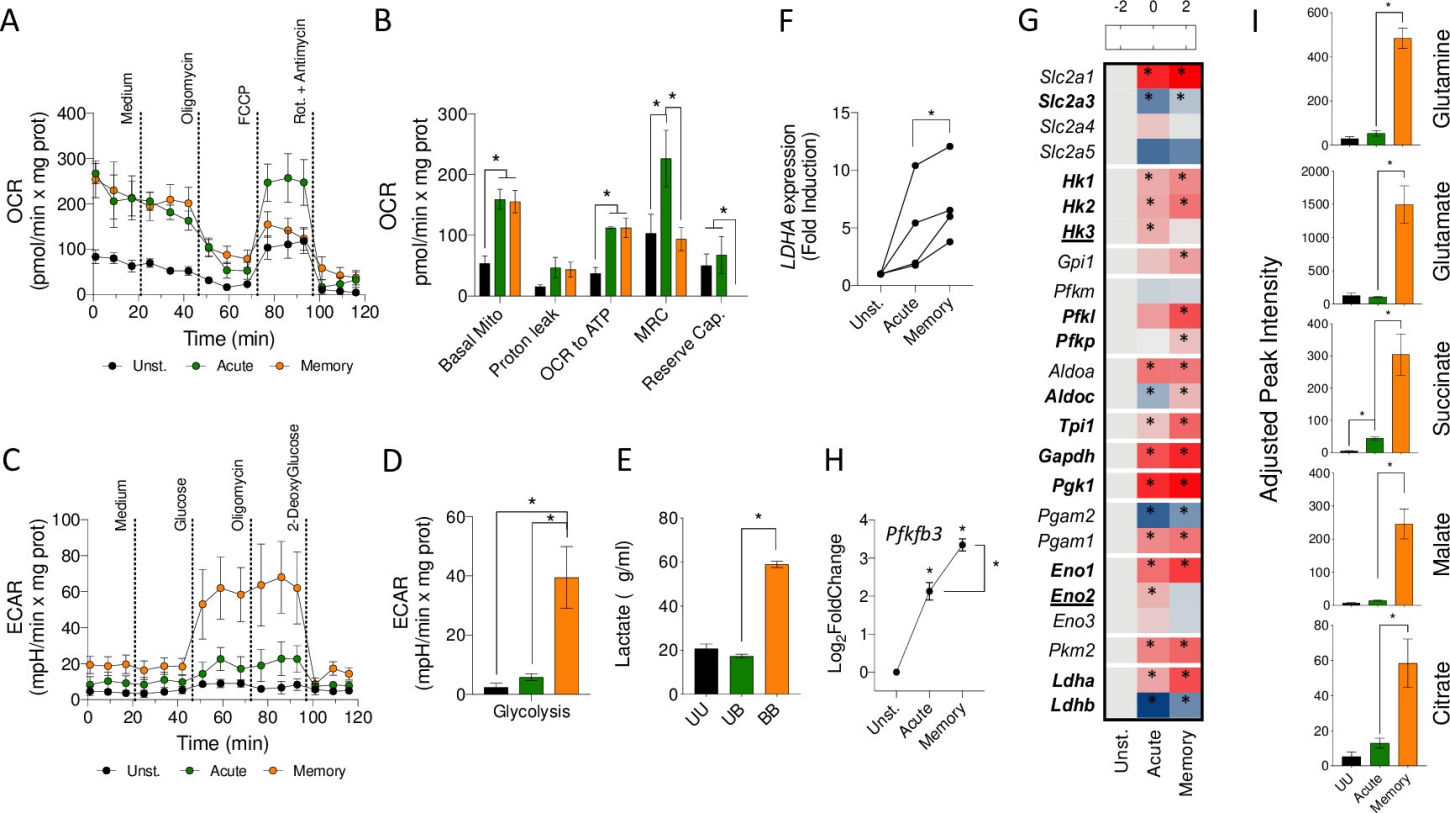
*B*. *burgdorferi*–induced metabolic changes in memory macrophages. Normalized OCR (A and B) and ECAR (C and D) of unstimulated (black), acute (green), and memory BMMs (orange). (E) Lactate production by unstimulated (black), acute (green), and memory murine macrophages (orange), measured in the cell supernatants. (F) *LDHA* expression in acute and memory human peripheral blood monocytes, as determined by real-time PCR relative to the gene, *RPLP0*. (G) Heat map showing the expression levels of glycolytic genes in acute and memory murine macrophages. The red color represents the up-regulation of genes, whereas down-regulation is indicated in blue. The asterisks indicate the genes that showed significantly different expression levels compared to unstimulated (Unst.) cells. Genes in bold represent those significantly increased in memory cells compared to acutely stimulated macrophages. Bold, underlined genes represent those significantly decreased in memory cells compared to acutely stimulated BMMs. (H) *Pfkfb3* gene expression changes in acute and memory macrophages in response to *B*. *burgdorferi* stimulation. (I) Intermediate metabolites of the TCA cycle in unstimulated (UU, black bars), acute (green bars), and memory (orange bars) BMMs. *, *p* < 0.05. The data underlying the graphs in Fig 5 can be found in S5 Data and GSE125503. BMM, bone marrow-derived macrophage; ECAR, extracellular acidification rate; FCCP, carbonyl cyanide-4-(trifluoromethoxy)phenylhydrazone; OCR, oxygen consumption rate; PCR, polymerase chain reaction; Rot, rotenone; TCA, tricarboxylic acid cycle.

IRF4 is a transcriptional regulator associated with metabolic changes in macrophages [44]. Furthermore, IPA identified the HIF1α pathway as up-regulated in memory macrophages compared to acutely stimulated cells in our RNA-seq data (Table 1), suggesting that memory development was accompanied by metabolic changes. We, therefore, analyzed the metabolic status of memory macrophages by Seahorse. Memory macrophages showed similar oxygen consumption rates (OCRs) than acutely stimulated BMMs (Fig 5A and 5B), although with lower maximal respiratory (MRC) and reserve capacities. In contrast, the glycolytic capacity of memory macrophages was significantly higher than in acutely stimulated cells (Fig 5C and 5D) and correlated with the presence of increased levels of lactate in the memory supernatants (Fig 5E) as well as the increased expression of the gene encoding the enzyme lactate dehydrogenase in both murine (log_2_ fold induction = 1; Padj = 2.1 × 10^−18^, between memory and acute macrophages) and human cells (Fig 5F). Moreover, the increased glycolytic capacity correlated with the augmented expression of several glycolytic genes (Fig 5G) and the higher expression levels of *Pfkfb3*, the gene encoding the positive regulator of glycolysis, fructose-2,6-bisphosphatase 3 [45], in memory macrophages (Fig 5H). On the other hand, the quantitative analysis by gas chromatography–mass spectrometry (GC–MS) of several intermediate metabolites of the TCA showed highly increased levels of glutamine, glutamate, succinate, citrate, and malate in memory cells compared to acutely stimulated macrophages (Fig 5I). These data confirm previous reports [24,27] and suggested increased glutaminolysis in restimulated macrophages and the conversion of malate to pyruvate to induce higher levels of lactate.

**Table 1:** Upstream pathways regulated in memory macrophages compared to acutely stimulated cells.

Upstream regulator	Expression log ratio	Activation z score	*p*-value of overlap
Prostaglandin E2		3.843	5.92E-29
*Il6*	1.569	3.7	5.67E-35
Cg		3.294	4.86E-14
***Hif1a***	0.685	3.223	2.74E-15
*Stat3*	0.479	3.165	1.63E-25
Ca^2+^		3.156	1.1E-10
FGF2		3.084	1.98E-15
Forskolin		3.045	4.73E-19
Mek		3.04	2.76E-09
*Cd38*	1.002	3.003	7.84E-07
Androgen		−2.646	3.86E-05
NS-398		−2.673	1.11E-7
Silibinin		−2.742	1.26E-03
CD3		−2.811	3.3E-15
Salirasib		−2.813	1.26E-04
*Stk11*	0.089	−2.88	9.04E-04
AP5		−2.882	2.09E-05
EGTA		−2.961	2.91E-07
Linsidomine		−2.961	5.92E-07
H89		−3.285	1.01E-13

Pathways activated (z-score >2) and repressed (z-score < −2) were identified using the IPA tool. The table represents the 10 most up-regulated and 10 most repressed upstream regulator pathways. The full list is provided in S5 Table.

IPA, Ingenuity Pathway Analysis.

### Glycolysis inhibition modulates the response of murine macrophages and inflammation during Lyme borreliosis

**Fig 6:**
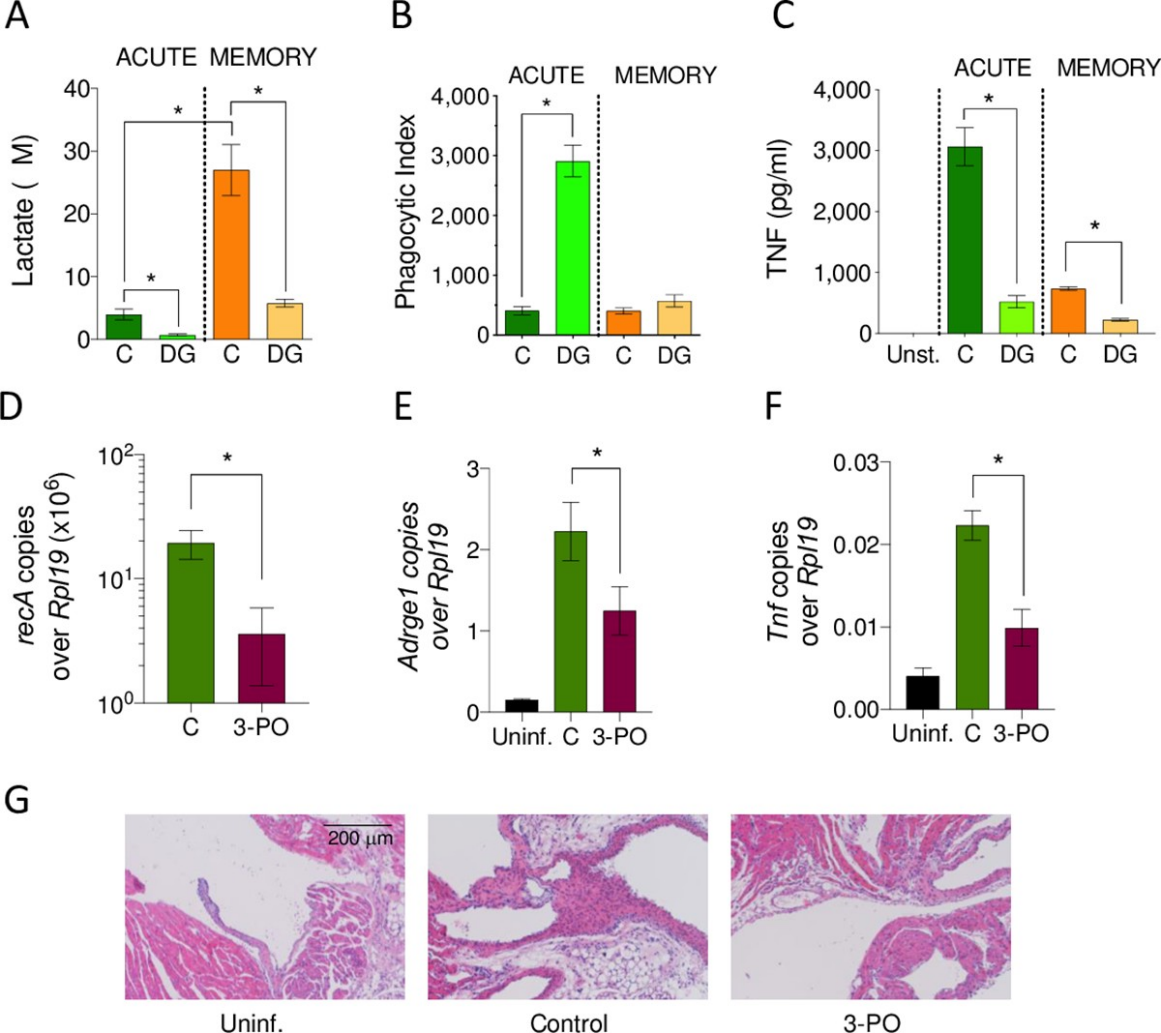
Glycolysis inhibition regulates the response of murine macrophages and modulates inflammation during infection. (A) Lactate production, (B) phagocytosis, and (C) TNF induction by *B*. *burgdorferi* in acute and memory macrophages in the presence or absence of the glycolysis inhibitor, 2-DG. (D) *B*. *burgdorferi* burdens in the heart of 3-PO-treated, infected mice (purple bar) compared to control-treated animals (green bar), as determined by DNA real-time PCR using primers specific for *recA* and relative to the housekeeping gene, *Rpl19*. (E) Macrophage infiltration and (F) *Tnf* gene expression in the heart of 3-PO-treated, infected mice (purple bars), control-treated infected animals (green bars) and uninfected controls (black bars) as determined by cDNA real-time PCR relative to *Rpl19*. The mice were infected for 2 weeks and then treated with the inhibitor for 3 more weeks. The results shown represent 5 mice per group. *, *p* < 0.05. (G) Hematoxylin and eosin staining of representative heart sections from mice infected with *B*. *burgdorferi* and treated with the glycolysis inhibitor, 3-PO. The data underlying the graphs in Fig 6 can be found in S6 Data. 2-DG, 2-deoxyglucose; 3-PO, 3-(3-pyridinyl)-1-(4-pyridinyl)-2-propen-1-one; PCR, polymerase chain reaction; TNF, tumor necrosis factor; Uninf.,uninfected.

We next assessed whether the inhibition of the glycolytic output of macrophages would affect the phagocytic capacity of these cells and/or their inflammatory output. Unstimulated and 48-hour activated BMMs were stimulated with *B*. *burgdorferi* in the presence of the glucose analogue, 2-deoxyglucose (2-DG). In both acute and memory macrophages, the use of 2-DG reduced the production of lactate (Fig 6A). The inhibition of glycolysis resulted in a significant increased ability of naive macrophages to phagocytose *B*. *burgdorferi* (Fig 6B), accompanied by decreased levels of TNF (Fig 6C). The inhibition of glycolysis did not, however, affect the phagocytic capacity of memory macrophages (Fig 6B), although it resulted in lower production of TNF (Fig 6C).

In order to assess whether the inhibition of glycolysis in vivo during infection with *B*. *burgdorferi* would affect the levels of bacteria in the heart (where macrophage infiltration is most evident) or their inflammatory status, we treated the infected animals with the PFKFB3 inhibitor, 3-(3-pyridinyl)-1-(4-pyridinyl)-2-propen-1-one (3-PO) [46]. The mice were treated after 2 weeks of infection, at the peak of disease, for a period of 3 weeks. The treatment with 3-PO resulted in significant reduced levels of spirochetes (Fig 6D), macrophage infiltration (Fig 6E), and *Tnf* expression (Fig 6F), compared to control mice, which could also be assessed at the histological level (Fig 6G). The reduction in spirochetal levels was not likely the result of a direct effect of 3-PO on spirochetal survival, since the growth of *B*. *burgdorferi* in the presence of 0.1 or 1 μM of the glycolysis inhibitor was not affected after 5 days in culture (S6 Fig). These results show that the inhibition of glycolysis in vivo during an ongoing infection with *B*. *burgdorferi* results in better control of infection, lower macrophage infiltration, and reduced inflammation in the heart.

### Inhibition of glycolysis results in the reversion of the macrophage memory phenotype and cardiac transcriptional changes induced by infection

**Fig 7:**
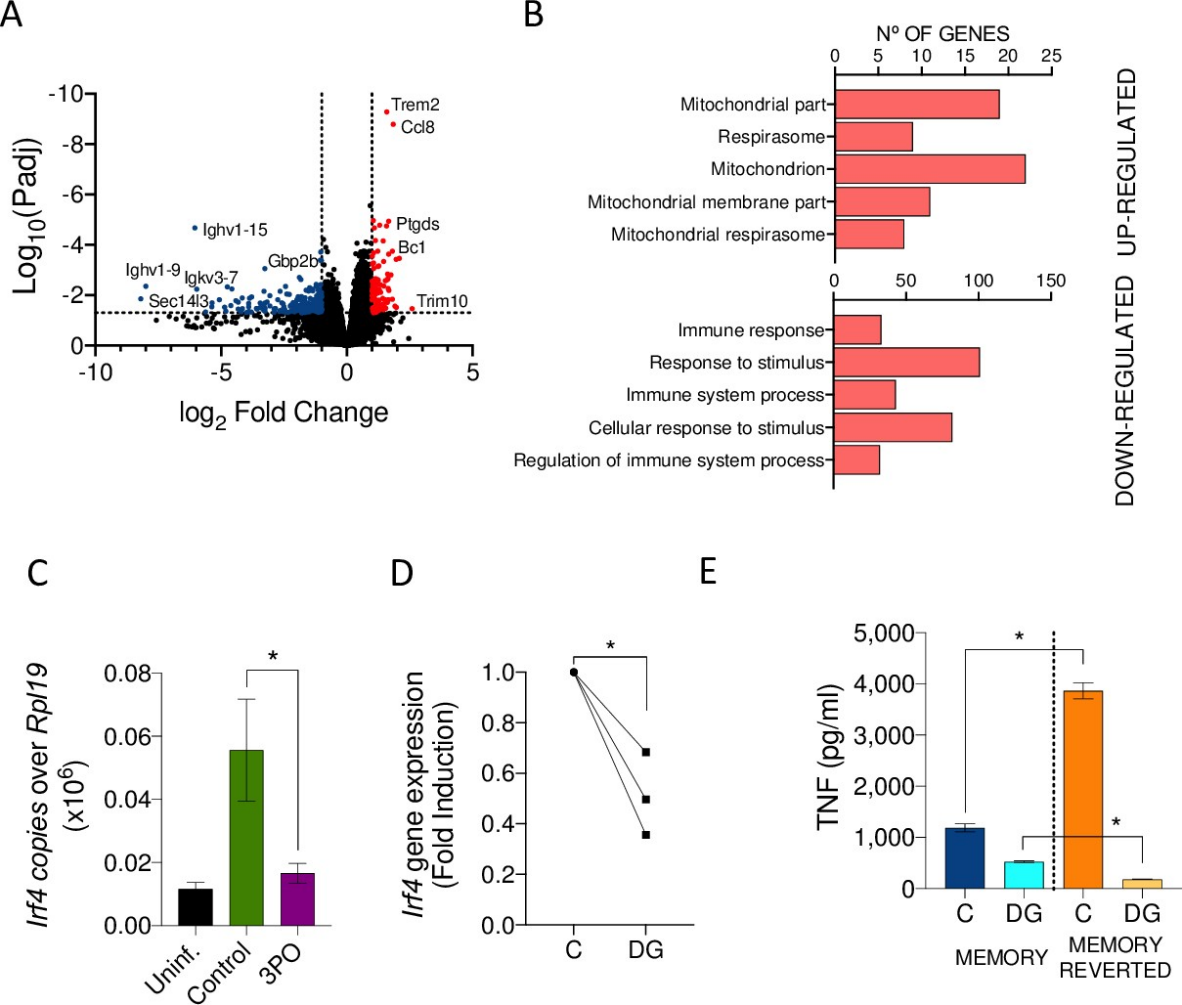
Inhibition of glycolysis reverts the *Irf4*-induced memory phenotype in murine macrophages and during Lyme borreliosis. (A) Volcano plot representing genes differentially expressed in the hearts of mice infected and treated with 3-PO compared to infected controls. The red dots represent genes up-regulated (110), while the blue dots correspond to down-regulated genes (232) in 3-PO treated animals determined by the absolute value of log2 fold induction (= 1) and Padj < 0.05. (B) Bar graphs showing the number of genes up- (top) and down-regulated (bottom) in infected animals treated with 3-PO compared to infected controls. The legend indicates GO-BP. (C) *Irf4* gene expression in the hearts of mice infected and treated with 3-PO starting day 14 of infection (purple bar), compared to control-treated infected mice (green bar) and uninfected controls (black bar). Gene expression levels were determined by real-time PCR relative to *Rpl19*. Groups of 5 mice were analyzed. *, Student *t* test, *p* < 0.05. (D) *Irf4* expression by memory macrophages treated with 2-DG during restimulation (20 hours) with *B*. *burgdorferi* compared to untreated controls. Results were obtained from 3 independent mice. *, Student *t* test, *p* < 0.05. (E) TNF production by control-treated memory macrophages (MEMORY) and memory macrophages pretreated with 2-DG for 20 hours (MEMORY REVERTED). Both memory and memory reverted macrophages were stimulated with *B*. *burgdorferi* for 20 hours in the absence (C) or presence of 2-DG. The results represent the average ± SE of 4 independent mice. The data underlying the graphs in Fig 7 can be found in S7 Data and GSE152168. 2-DG, 2-deoxyglucose; 3-PO, 3-(3-pyridinyl)-1-(4-pyridinyl)-2-propen-1-one; GO-BP, Gene Ontology Biological Process; PCR, polymerase chain reaction; SE, standard error; TNF, tumor necrosis factor.

In order to identify changes in gene expression at the tissue level as a consequence of the treatment with the glycolysis inhibitor, 3-PO, we performed a transcriptomic analysis of the hearts of the mice treated with the inhibitor and their control-treated counterparts. The comparison of both conditions showed 110 genes up-regulated in infected mice treated with 3-PO, while 232 genes were significantly reduced (Fig 7A). Genes up-regulated by the treatment with 3-PO corresponded to those associated with mitochondria (Fig 7B), indicating that the treatment had, at least partially, reverted the effect of infection in this organelle at the whole tissue level. On the other hand, the genes down-regulated in the hearts of the 3-PO mice included those associated with immunity and the response to stimulus (Fig 7B), as further observed in the analysis of *Tnf* gene expression levels (Fig 6F).

The analysis of *Irf4* expression showed a significant reduction in mice treated with 3-PO (Fig 7C). These results suggested that the inhibition of glycolysis during an ongoing infection with *B*. *burgdorferi* results in the reversion of the memory-induced *Irf4* expression, while affecting the infiltration of macrophages and the ensuing inflammatory response. Indeed, the inhibition of glycolysis in memory macrophages in vitro resulted in the decreased expression of *Irf4* (Fig 7D) and increased TNF production upon a subsequent stimulation with *B*. *burgdorferi* (Fig 7E). Interestingly, the presence of 2-DG during a successive encounter with the spirochete in previously inhibited memory macrophages dramatically diminished TNF production (Fig 7E). These data suggest that the inhibition of glycolysis results in the reversion of the memory phenotype, leading to increased susceptibility to further treatment with the glycolysis inhibitor, including the increased ability to phagocytose *B*. *burgdorferi*. These results also highlight the relevance of *Irf4* regulation as a marker of memory generation.

## Discussion

**Fig 8:**
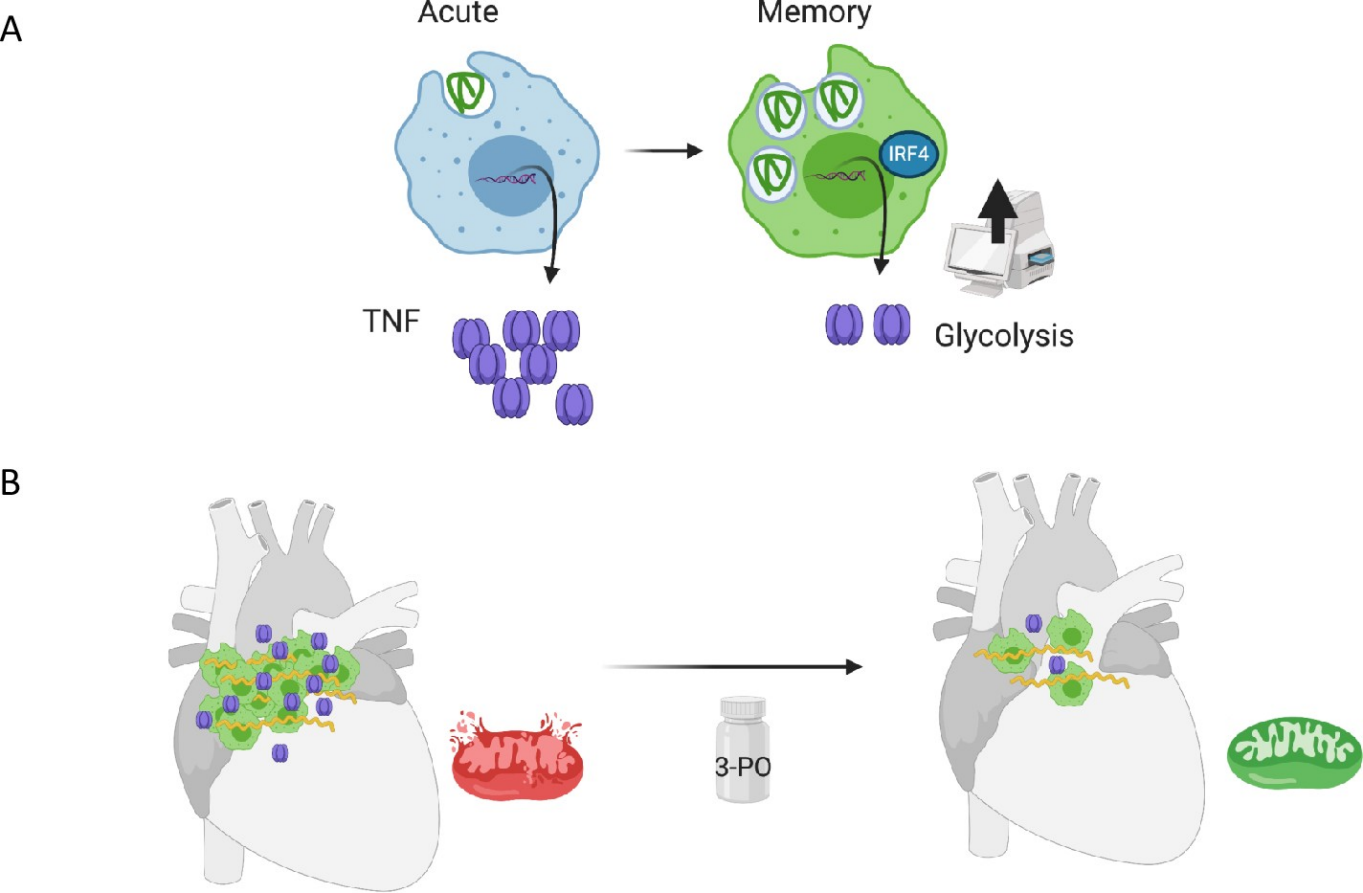
Cartoon model of the generation of innate memory responses to *B*. *burgdorferi* in vitro and in vivo. (A) Memory macrophages show increased binding of the spirochete, their internalization, and decreased production of TNF compared to acutely stimulated cells. Memory cells also show increased expression of *Irf4* and augmented glycolysis. (B) Cardiac infection with *B*. *burgdorferi* results in the infiltration of macrophages with increased expression of *Irf4* and the down-regulation of mitochondrial proteins and gene expression. The inhibition of glycolysis during infection with the PFKFB3 inhibitor, 3-PO, results in decreased bacterial burdens, reduced macrophage infiltration, and lower expression of *Tnf*. The transcriptomic analysis of 3-PO-treated, infected hearts show increased expression of mitochondrial genes. The art work was created with BioRender.com. 3-PO, 3-(3-pyridinyl)-1-(4-pyridinyl)-2-propen-1-one; TNF, tumor necrosis factor.

The heart is the most energy-demanding organ in the human body, with mitochondrial oxidative phosphorylation being responsible for nearly all of the ATP production in adult mammalian hearts [12]. Therefore, abnormalities in mitochondrial function are known causes of cardiomyopathies, arrhythmias, abnormalities of the conduction system, ischemia-reperfusion injury, heart failure, and aging among others [16–18]. Inflammation is known to induce mitochondrial damage by oxidation, nitration or nitrosation, leading to energetic supply failures [47]. Among the tissues affected during infection with *B*. *burgdorferi*, the heart is one of the targets of inflammation with functional consequences at the conduction level. We now describe the overall tissue effects on the transcriptional and proteomic status of mice infected with the spirochete and show that mitochondria are a main organelle affected by infection. We also show that the manipulation of long-term macrophage responses has beneficial effects against inflammation and the mitochondrial deficit that results from infection (Fig 8).

Innate immune memory has been defined as the long-term modulation of monocyte/macrophage responses upon an initial encounter with primarily single pathogen-associated molecular patterns (PAMPs) or dead bacteria [23,25]. These responses have been studied exclusively in the context of pro-inflammatory cytokine induction [23]. Here, we define the long-term consequences of the interaction of macrophages with live *B*. *burgdorferi*, a spirochete able to establish persistent infections in mammals. Because the response of macrophages to live and dead spirochetes is both quantitatively and qualitatively distinct [30,48] and involves the capacity of these cells to eliminate bacteria [29,49], we provide a new perspective on the concept of innate immune memory. The continuous exposure to *B*. *burgdorferi* induces an increased capacity to internalize the spirochete, albeit with a diminished production of pro-inflammatory cytokines. The induction of innate memory responses to the spirochete seem to constitute an advantage to the host, since it results in the control of the spirochete and a reduced inflammatory output. However, *B*. *burgdorferi* is still able to persist under these conditions probably due to their recognized ability to subvert not only the adaptive immune system, but also innate immune responses [5]. Overall, our data show that a further modulation of ongoing innate immune memory responses can shift the balance toward a better control of the spirochete and/or the ensuing inflammatory response.

We have pinpointed the changes associated with the long-term exposure to the spirochete to a single gene, *Irf4*, which regulates the expression of cytokines such as TNF and IL-6. *Irf4* is regulated by 3 transcription factors, SpiB, MAFK, and MZF1, which control the expression of several differentially expressed genes in memory and naïve macrophages. IRF4 is a transcriptional regulator that has also been involved in the negative control of MyD88-induced pro-inflammatory cytokine production, through competition with IRF5 [50,51]. Importantly, those genes directly associated with the interaction between MyD88 and IRF5, including *Tnf*, *Il6*, and *Il12b* [50], showed reduced transcription levels in cells with higher expression of *Irf4*. Overall, these results suggest that in *B*. *burgdorferi*–specific memory macrophages, the up-regulation of IRF4 may interfere with MyD88-IRF5-derived gene expression.

The long-term responses induced by macrophage exposure to *B*. *burgdorferi* are also characterized by metabolic changes that are partially consistent with innate memory responses, including an increased glycolytic output [52]. Indeed, increased *PFKFB3*, *HK2*, and *LDHA* gene expression has been observed in Lyme borreliosis patients, as well as increased levels of serum lactate [53]. Therefore, our results support the metabolic switch in infected patients and pinpoint these changes to monocyte/macrophages, albeit they do not discount metabolic shifts in other immune cells. *B*. *burgdorferi* also induces increased glutaminolysis in memory macrophages, as well as the repression of genes that lead to fatty acid synthesis (*Acsl1*: log_2_FoldInduction = −0.82; adjusted *p*-value = 1.01E-13; *Acss2*: log_2_FoldInduction = −1.58; adjusted *p*-value = 2.01E-22) [27]. Our results also imply the increased production of malate, among other components of the TCA, which can be shuttled to its conversion to pyruvate and the increased production of lactate by the augmented expression of the enzyme, lactate dehydrogenase. Importantly, we show that the inhibition of glycolysis in vivo during an ongoing infection with *B*. *burgdorferi* results in decreased cardiac inflammatory responses, in spite of a lower sensitivity of memory macrophages inhibited in vitro with 2-DG. The inhibition of glycolysis results in the down-regulation of *Irf4* both in vitro and in vivo, suggesting a decreased memory phenotype of the infiltrating macrophages. At the same time, both macrophage infiltration and TNF production are diminished as a consequence of the treatment. Because *B*. *burgdorferi*–specific innate memory cells are less susceptible to glycolysis inhibition, our data suggest the reversion of the memory phenotype, which, in turn, renders the cells more susceptible to the ongoing presence of the inhibitor, resulting in an IRF-4-independent reduction of pro-inflammatory cytokine production. At the same time, the augmented capacity of the cells to control spirochetemia in the heart results in a decrease in the number of bacteria, which eventually may provoke decreased macrophage infiltration.

In human Lyme disease, the most common cardiac complication is atrioventricular block, which has been associated with the presence of spirochetes in the cardiac tissue and the ensuing inflammatory response [54,55], although histopathological data are scarce [4]. Similarly, data associating murine infection with conduction disturbances are not extensively available. Saba and colleagues described the electrophysiological consequences of infection with *B*. *burgdorferi* in C3H/J mice of different ages and sexes [56]. They observed conduction abnormalities that reversed in parallel with the resolution of inflammation. Similarly, Armstrong and colleagues performed electrocardiographs in infected C3H and B6 mice over time, noting higher prevalence of episodes of bradycardia and tachycardia in C3H mice than in B6 animals [57]. Recently, Hulsmans and colleagues have described a critical role of cardiac resident macrophages, which are present in the AV node, in the modulation of the electrical activity of cardiomyocytes through physical contact [58]. They argue that a change in the phenotype of these resident cells may be responsible for conduction abnormalities observed during diverse pathologies, including those that cause inflammatory responses [58]. It is tempting to speculate that a change in the homeostatic macrophage environment under infection-induced inflammatory conditions may affect the ability of resident macrophages to control the normal electric activity of the heart and that the modulation of macrophage activity can have functional beneficial consequences in the electric activity of the cardiac tissue. Further functional work should provide evidence of whether this is the case during Lyme borreliosis.

Overall, our data show that the stimulation of macrophages with *B*. *burgdorferi* is dynamic and while affecting those pathways already described [33], is modulated by the continuous stimulation with the spirochete. They also show that the memory phenotype is susceptible to be modified in an ongoing infection, resulting in an overall decrease in inflammation.

## Materials and methods

### Experimental animals

C57Bl/6 (B6) mice were purchased from Charles River Laboratories (Lyon, France) and bred in the Animal Facility at CIC bioGUNE, where they were socially housed under a 12-hour light/dark cycle. All murine infections were performed in 7- to 8-week old animals. BMMs were obtained from 6- to 12-week-old mice. Both males and females were used throughout. All the procedures were approved by the competent authority (Diputación de Bizkaia) under European and Spanish directives. CIC bioGUNE’s Animal Facility is accredited by AAALAC International.

### Murine bone marrow-derived macrophages (BMMs)

Bone marrow cells were obtained from the femoral and tibial shafts and subjected to erythrocyte lysis with ACK buffer (NH_4_Cl 150 mM; KHCO_3_ 10 mM; Na_2_EDTA 0.1 mM). The cells were incubated in non-treated 100 mm × 15 mm plates (Thermo Fisher Scientific, Alcobendas, Spain) for 6 days at 37°C in DMEM (Thermo Fisher Scientific) supplemented with 10% fetal bovine serum (FBS) (Thermo Fisher Scientific) and 1% penicillin/streptomycin (Thermo Fisher Scientific) plus 30 ng/ml of M-CSF (Miltentyi Biotech, Pozuelo de Alarcón, Spain). The medium was changed every 3 days. Nonadherent cells were discarded, and the differentiated macrophages were scraped in PBS (Thermo Fisher Scientific) + 1% FBS, counted, and seeded at the appropriate concentrations.

### Human peripheral blood monocyte purification and macrophage differentiation

Human monocytes were purified from buffy coats of healthy donors by positive selection using a CD14 purification kit (Miltenyi Biotech). Peripheral blood monocytic cells were first isolated by ficoll density centrifugation (GE Healthcare, Madrid, Spain) at 400 xg for 30 minutes without braking. The monocyte layer was recovered, washed, and processed according to the manufacturer’s protocol. Monocytes were cultured in RPMI (Thermo Fisher Scientific) supplemented with 10% FBS and 1% penicillin/streptomycin and rested overnight before stimulation. A total of 7 human samples were used, obtained after approval by the Basque Country’s Ethics Committee following the Helsinki convention. Donors signed an informed consent form and were anonymized to the authors.

Human monocyte-derived macrophages were generated as described [33]. Briefly, purified monocytes were incubated for 6 days in the presence of 50 ng/ml of human M-CSF (Miltenyi Biotec), with medium changes every 2 days. The differentiated macrophages were washed, counted, and stimulated as described above.

### Cell lines

HEK293FT and RAW 264.7 cells were cultured in DMEM supplemented with 10% FBS and 1% penicillin/streptomycin. A low passage number (0 to 20) of both cell lines were maintained in 100 mm × 20 mm plates for adherent cells (Sarstedt, Nümbrecht, Germany).

### Bacteria

*B*. *burgdorferi* s.s. strain 297, clone Bb914 [59] and strain B31, clone 5A15 [60] were used throughout. The spirochetes were grown in BSK-H medium (Sigma-Aldrich, Madrid, Spain) in 5-ml tubes at 34°C and counted in a dark field microscope (Zeiss, Munich, Germany).

### Murine infections

Mice were subcutaneously infected in the midline of the back with 10^5^ *B*. *burgdorferi* B31 clone 5A15, using a solution of 2× washed mid-log phase bacteria in PBS at a concentration of 10^6^ spirochetes/ml [39]. At killing, the hearts were cut in half through bisections across the atria and ventricles to isolate DNA and RNA using the AllPrep DNA/RNA/miRNA Universal Kit (Qiagen, Las Rozas de Madrid, Spain) following the manufacturer’s recommendations. Bacterial burdens were measured from heart DNA by quantitative polymerase chain reaction (qPCR) targeting *recA* relative to the murine gene, *Rpl19*. RNA was reverse-transcribed with M-MLV (Thermo Fisher Scientific) and used to perform real-time PCR to determine the expression levels of *Tnf*, *Adgre1*, *Itgam*, and *Irf4* relative to *Rpl19*, as before. The isolated RNA from infected hearts was also employed to perform comparative RNA-seq transcriptomics analysis.

After killing, blood was quickly obtained by intracardiac puncture to isolate RNA as follows: 200 μl of blood were vigorously mixed with 800 μl of TRIzol (Thermo Fisher Scientific), incubated at room temperature for 5 minutes and mixed with 200-μl chloroform (Sigma-Aldrich). The mixture was centrifuged at 12,000 xg for 15 minutes at 4°C and the upper layer was transferred to a new tube and precipitated with 500-μl isopropanol (Panreac AppliChem, Castellar del Vallès, Spain). The RNA was resuspended in 750-μl ethanol 75%, loaded into Nucleospin RNA columns (Nucleospin RNA kit, Macherey-Nagel, Düren, Germany), and eluted following the manufacturer’s recommendations. The expression levels of *Irf4* were determined relative to *Rpl19* by real-time RT-PCR from reverse-transcribed RNA.

### Tissue MALDI Imaging (MALDI-IMS) of infected hearts

The hearts of 3 week-infected mice and uninfected controls (4 each) were washed of excess blood, embedded in optimal cutting temperature (OCT) compound (Thermo Fisher Scientific), and stored at −80°C. The tissue was processed in 14-μm sections into Indium Tin Oxide coated slides in a microtome (Leica, Wetzlar, Germany) using an OCT-free setting with frozen water. OCT was removed with absolute ethanol, and the samples were included in frozen water, following a water inclusion procedure [61]. The samples were delipidized through serial passages of increasing concentrations of ethanol (70% to 100%) and dried in a vacuum chamber for 20 minutes. The hearts were then sprayed with a matrix composed of sinapinic acid (15 mg/ml) in 75% acetonitrile and 0.2% trifluoroacetic acid, using a Langartech robot [62], with a total of 10 layers.

The samples were subjected to MALDI-TOF in linear mode on a Bruker Daltonics Autoflex III MALDI-TOF/TOF (Bremen, DE) equipped with a SmartBeam Nd:YAG/355-nm laser, with 200-Hz pulses. Acquisition was performed using lineal geometry in the ranges of mass between 2,000 and 30,000 Th in positive mode. All instrumental parameters (delayed extraction, laser fluence, and detector gain) were optimized until the optimal signal/noise ratio was achieved along the spectrum. A total of 500 cumulated shots (random shooting) per spot were collected with 100-μm spacing on each selected section. Acquisition was done with FlexControl 3.0 (Bruker Daltonics). External calibration was performed in a unique point per section with ProtMix 1 and ProtMix 2 (Bruker Daltonics).

Data analysis was performed with the software package FlexImaging 3.0 and ClintProtTools 2.1 (Bruker Daltonics). Routinely, the numeric value of each detected mass was identified using the algorithm, Centroide, giving as results the average mass of each peak. Minimal treatment of each sample was performed using Savistky–Golay smoothing at 2 m/z and 5 cycles, as well as basal line correction using the algorithm TopHat and data reduction of 20 m/z.

### Proteomic analysis of *B*. *burgdorferi*–infected hearts

The hearts of 3 week-infected mice and uninfected controls (4 each) were soaked in PEB buffer (PBS 1X, 2 mM EDTA, 0.5% BSA) and transferred to 60-mm plates. The organs were washed to eliminate excess of blood and cleaned of conjunctive tissue and other impurities. The top third of each heart was cut and placed in 200 μl of CLB buffer (7 M urea, 2 M thiourea, and 4% CHAPS). The tissue was then dissociated using a pestle and motor mixer (VWR, Llinars del Vallés, Spain) and digested following the filter-aided FASP protocol described by Wisniewski and colleagues [63] with minor modifications. Trypsin was added to a trypsin:protein ratio of 1:10, and the mixture was incubated overnight at 37 ^o^C, dried out in a RVC2 25 Speedvac concentrator (Christ), and resuspended in 0.1% FA.

The equivalent of approximately 500 ng of each sample was submitted to liquid chromatography–mass spectrometry (LC–MS) label-free analysis. Peptide separation was performed on a nanoACQUITY UPLC System (Waters, Milford, Massachusetts, United States of America) online connected to an LTQ Orbitrap XL mass spectrometer (Thermo Fisher Scientific). An aliquot of each sample was loaded onto a Symmetry 300 C18 UPLC Trap column (180 μm × 20 mm, 5 μm (Waters)). The precolumn was connected to a BEH130 C18 column (75 μm × 200 mm, 1.7 μm (Waters), and equilibrated in 3% acetonitrile and 0.1% FA. Peptides were eluted directly into an LTQ Orbitrap XL mass spectrometer (Thermo Fisher Scientific) through a nanoelectrospray capillary source (Thermo Fisher Scientific), at 300 nl/min and using a 120-minute linear gradient of 3% to 50% acetonitrile. The mass spectrometer automatically switched between mass spectrometry (MS) and tandem mass spectrometry (MS/MS) acquisition in DDA mode. Full MS scan survey spectra (m/z 400 to 2,000) were acquired in the orbitrap with mass resolution of 30,000 at m/z 400. After each survey scan, the 6 most intense ions above 1,000 counts were sequentially subjected to collision-induced dissociation (CID) in the linear ion trap. Precursors with charge states of 2 and 3 were specifically selected for CID. Peptides were excluded from further analysis during 60 seconds using the dynamic exclusion feature.

Progenesis LC–MS (Waters) was used for the label-free differential protein expression analysis. One of the runs was used as the reference to which the precursor masses in all other samples were aligned to. Only features comprising charges of 2+ and 3+ were selected. The raw abundances of each feature were automatically normalized and logarithmized against the reference run. Samples were grouped in accordance to the comparison being performed, and an ANOVA analysis was performed. A peak list containing the information of all the features was generated and exported to the Mascot search engine (Matrix Science, London, United Kingdom). This file was searched against a Uniprot/Swissprot database consisting of *Mus musculus* and *Borrelia* entries, and the list of identified peptides was imported back to Progenesis LC–MS. Protein quantitation was performed based on the 3 most intense nonconflicting peptides (peptides occurring in only 1 protein), except for proteins with only 2 nonconflicting peptides. The significance of expression changes was tested at protein level, and proteins with an ANOVA *p*-value ≤ 0.05 were selected for further analyses. Ontological analysis were performed using STRING [64].

### 3-PO treatment during murine infection

Groups of 5 mice were infected and treated with 50 mg/kg 3-PO (Sigma-Aldrich) by intraperitoneal injection, twice per week during 3 weeks.

### In vitro stimulation with *B*. *burgdorferi*

BMMs were scraped, counted, and seeded at 1 × 10^6^ cells/well in 6-well plates (Thermo Fisher Scientific) and rested for 6 hours. Macrophages were then stimulated with *B*. *burgdorferi* according to the scheme depicted in S3A Fig. To account for the loss of cells due to stimulation with *B*. *burgdorferi* ([29,65], S7 Fig), the amount of cells was adjusted to obtain equivalent numbers after the first 48-hour stimulation. Human CD14^+^ monocytes and monocyte-derived macrophages were stimulated under the same conditions after an overnight resting period. Silenced RAW264.7 cells and BMMs were stimulated for 16 to 20 hours, and the supernatants were collected to measure TNF and lactate. In specific experiments, the glucose analogue, 2-DG (Sigma-Aldrich), was added at different time points at a final concentration of 0.5 mM.

### In vitro phagocytosis assays

Phagocytosis assays were performed in 12-well plates (Thermo Fisher scientific) using 3.5 × 10^5^ cells per well as previously described [66]. BMMs and RAW 264.7 cells were cultured in serum- and antibiotic-free DMEM for 1 hour. GFP expressing *B*. *burgdorferi* were added to the cells at a multiplicity of infection (MOI) of 25 and incubated at 4°C for 15 minutes followed by 37°C for 2 hours. The cells were then washed to eliminate surface bacteria and analyzed by flow cytometry in a BD FACS Canto II (BD Biosciences, San Agustín de Guadalix, Spain). Controls at 4°C were run in parallel to assess washing efficiencies. The data were analyzed using Flowjo version 10. The phagocytic index was calculated using the formula: % GFP cells (Test) × mean fluorescence intensity (MFI) (Test) − % GFP cells (4°C control) × MFI (4°C control) [42]. Binding experiments were performed at 4°C, followed by gentle washing to avoid the elimination of the bound bacteria.

### *Irf4* gene silencing in RAW264.7 cells and BMMs

Lentiviral particles containing short hairpin RNA (shRNA) targeting *Irf4* (Sigma-Aldrich) were generated using a third-generation lentivirus vector with a conditional packaging system [67,68]. The 3 × 10^6^ HEK293FT cells were seeded in 100 mm × 20 mm plates and transfected by the dropwise method using CaPO_4_ and a mixture of 5-μg lentiviral plasmids (pRSV-Rev, pMDLg/pRRE, and pCMV-VSV-G; Addgene) plus 5 μg of the sh*Irf4* plasmid, preincubated for 30 minutes in the presence of CaCl_2_ (Sigma-Aldrich) and HBS (25mM HEPES; 140Mm NaCl; 5mM KCl; 0.75mM Na2HPO4; 6mM glucose, pH 7.05). Culture supernatants were harvested 48 and 72 hours posttransfection, incubated with Lenti-X concentrator (Takara, Saint-Germain-en-Laye, France), and the lentiviral particles were concentrated by centrifugation at 1,500 xg for 45 minutes at 4°C. Lentiviral pellets were resuspended in DMEM supplemented with 10% FBS and 1% penicillin/streptomycin plus 8 μg/ml of protamine sulfate (Sigma-Aldrich). RAW264.7 cells were infected 2 consecutive days followed by incubation with puromycin at 3 μg/ml (Sigma-Aldrich) to produce stable lines. BMMs were infected with lentiviral particles at days 3 and 5 of the differentiation process. The controls used were cells infected with lentiviral particles containing the empty vector, PLKO.1. The level of silencing relative to *Gapdh* was determined by real-time PCR, using the primers shown in S4 Table.

### Confocal microscopy

The 1.5 × 10^5^ BMMs were cultured in 16-mm cover slips (Marienfeld Superior, Lauda-Königshofen, Germany) within 24-well plates (Cultek, Madrid, Spain). The cells were stimulated with *B*. *burgdorferi* or left unstimulated for 48 hours and washed. The macrophages were then incubated with *B*. *burgdorferi* Bb914 at an MOI of 25 for 1 hour at 4°C, washed, and fixed with 4% paraformaldehyde (Sigma-Aldrich) for 20 minutes. The cells were then permeabilized with 0.3% Triton X-100 (VWR) and stained with rhodamine-labeled phalloidin and DAPI for 10 minutes at 37°C (Thermo Fisher Scientific). After extensive washing with PBS, the cells were mounted using the Prolong Gold Antifade mounting reagent (Thermo Fisher Scientific). The images were obtained employing a Leica TCS SP8 confocal system (Leica Microsystems, Madrid, Spain).

### Cytokine ELISA

The levels of murine and human TNF and murine IL-6 in the restimulation supernatants were determined by capture ELISA using the Mouse TNF ELISA Set II, the Mouse IL-6 ELISA Set (BD Biosciences), and the human TNF ELISA Set (BD Biosciences), following the manufacturers’ instructions.

### RNA isolation

Total RNA from RAW264.7 cells and BMMs were isolated using the NucleoSpin RNA kit (Macherey-Nagel). The quantity and quality of the RNAs were assessed using the Qubit RNA Assay Kit (Thermo Fisher Scientific) and RNA Nano Chips in a 2100 Bioanalyzer (Agilent Technologies, Santa Clara, California, USA), respectively.

### RNA-seq transcriptomics

Libraries were prepared using either the TruSeq RNA Sample Preparation Kit v2 or the TruSeq mRNA Library Prep (Illumina, San Diego, California, USA) following the instructions from the manufacturer. Single-read 50-nucleotide (nt) sequencing of pooled libraries was carried out in HiScanSQ or HiSeq2500 platforms (Illumina). The quality control of the sequenced samples was performed using the FASTQC software (www.bioinformatics.babraham.ac.uk/projects/fastq). Reads were mapped against the mouse (mm10) reference genome using Tophat [69] accounting for spliced junctions. The resulting BAM alignment files for the samples were then used to generate a table of raw counts by Rsubread [70]. The raw counts table was the input for the differential expression (DE) analysis, carried out by DESeq2 [71], to identify differentially expressed genes among the different conditions. GO enrichment was tested using the clusterProfiler bioconductor package [72], the Panther Database [73], and DAVID (https://david.ncifcrf.gov) [74,75]. Transcriptomics data were also analyzed using Qiagen’s IPA (Qiagen). Motif enrichment analysis was performed using the HOMER software [43] for motif discovery, using the findMotifs.pl script. Motifs of lengths 8, 10, and 12 nt were considered in this analysis.

### Real-time PCR

RNA was reverse transcribed using M-MLV reverse transcriptase (Thermo Fisher Scientific). Real-time PCR was then performed using the PerfeCTa SYBR Green SuperMix low ROX (Quantabio, Beverly, Massachusetts, USA) on a QuantStudio 6 Real-Time PCR System (Thermo Fisher Scientific). Fold induction of the genes was calculated relative to *Rpl19* using the 2^−ΔΔ^Ct method. The primers used are listed in S4 Table.

### Metabolic assays

The OCR and extracellular acidification rate (ECAR) were measured in differentially stimulated BMMs employing an XF24 extracellular flux analyzer (Agilent Technologies). Unstimulated (4 × 10^5^) and *B*. *burgdorferi*–stimulated cells (2 × 10^5^) were seeded per well in a Cell-Tak coated plate (BD Biosciences), and the measurements were normalized to cellular protein amount. For ECAR determination, the cells were previously plated in XF Seahorse medium with 4 mM glutamine and 10 mM pyruvate, while for the mitochondrial stress test, the cells were plated in medium containing 4 mM glutamine, 10 mM pyruvate, and 25 mM glucose. After 1 hour at 37°C without CO2, 3 baseline OCR and ECAR measurements were performed. For glycolysis determination, ECAR was measured at baseline and after sequentially adding glucose (25 mM), Oligomycin (1 μM), and 2-DG (50 mM). In parallel experiments, OCR was determined at baseline and after sequentially adding oligomycin, FCCP, and antimycin/rotenone at 1 μM. Lactate production was measured from stimulation supernatants using the Lactate Assay (Sigma-Aldrich), L-lactate TRINDER liquid (Biochemical Enterprise, Milano, Italy), and Lactate-Glo™ Assay kits (Promega, Alcobendas, Spain). Luminescence assays were performed in white opaque 96-microwell plates (PerkinElmer, Tres Cantos, Spain) by diluting 1/40 the supernatants following the manufacturers’ recommendations. Light signal was read in a Veritas Microplate Luminometer (Turner BioSystems, Sunnyvale, California, USA).

### Determination of metabolic intermediaries of the TCA metabolism

The levels of glutamine, glutamate, malate, citrate, and succinate were determined in BMMs by liquid chromatography tandem mass spectrometry (LC–MS/MS). Cellular pellets were homogenized in 500 μl of ice-cold extraction liquid (ice cold methanol/water (50/50%v/v) and 1 μM stable labeled 13CD3-methionine (methionine-SL) as internal standard) with a tissue homogenizer (FastPrep) in one 30-second cycle at 6,000 rpm. Subsequently, 400 μl of the homogenate were shaken at 1,400 rpm for 30 minutes at 4°C. The samples were then centrifuged for 15 minutes at 13,000 rpm and 4°C. Moreover, 100 μl of the aqueous phase were placed at −80°C for 20 minutes, followed by evaporation in a Speedvac during approximately 3 hours. The resulting pellets were resuspended in 100 μl water/acetonitrile (MeCN) (40/60 v/v). Samples were measured with a UPLC system (Acquity, Waters) coupled to a time-of-flight mass spectrometer (ToF MS, SYNAPT G2, Waters). A 2.1 × 100 mm, 1.7 μm BEH amide column (Waters), thermostated at 40°C, was used to separate the analytes before entering the MS. Mobile phase solvent A (aqueous phase) consisted of 99.5% water, 0.5% FA, and 20 mM ammonium formate while solvent B (organic phase) consisted of 29.5% water, 70% MeCN, 0.5% FA, and 1 mM ammonium formate. In order to obtain a good separation of the analytes the following gradient was used: from 5% A to 50% A in 2.4 minutes in curved gradient (#8, as defined by Waters), from 50% A to 99.9% A in 0.2 minutes constant at 99.9% A for 1.2 minutes, back to 5% A in 0.2 minutes. The flow rate was 0.250 ml/min and the injection volume 2 μl. After every 6 injections, QC low and QC high sample was injected. The MS was operated in positive and negative electrospray ionization, depending on analyte, in full scan mode. The cone voltage was 25 V, and capillary voltage was 250 V. Source temperature was set to 120°C and capillary temperature to 450°C. The flow of the cone and desolvation gas (both nitrogen) were set to 5 L/h and 600 L/h, respectively. A 2 ng/mL leucine enkephalin solution in water/acetonitrile/formic acid (49.9/50/0.1%v/v/v) was infused at 10 μl/min and used for a lock mass which was measured each 36 seconds for 0.5 seconds. Spectral peaks were automatically corrected for deviations in the lock mass.

### Ethics statement

All work performed with animals was approved by an Órgano Habilitado (Comité de Bioética y Bienestar Animal, CBBA/IACUC, at CIC bioGUNE) and the competent authority (Diputación de Bizkaia) following European and Spanish regulations, under the following protocols: P-CBG-CBBA-0915 and P-CBG-CBBA-0917.

Human samples were obtained after approval by the Basque Country’s Ethics Committee following the Helsinki convention. Donors signed an informed consent form and were anonymized to the authors.

### Quantification and statistical analysis

The results are presented as the means ± standard error (SE). Significant differences between means were calculated with the Student *t* test. Multiple comparisons were analyzed by ANOVA, followed by pairwise comparisons. A *p*-value < 0.05 was considered significant. All statistical calculations were performed with GraphPad Prism version 8.

### Data and software availability

The accession number for RNA-seq data from murine *B*. *burgdorferi*–infected hearts is NCBI GEO: GSE152168.

The accession number for the RNA-seq data of BMMs differentially stimulated with *B*. *burgdorferi* is NCBI GEO: GSE125503.

The accession number for the proteomic analysis of *B*. *burgdorferi*–infected mouse hearts is PXD019605.

The MALDI-IMS data are included in S1 Supporting Information.

## Supporting information

S1 Fig MALDI-IMS analysis of cardiac tissue from B. burgdorferi–infected and noninfected controls. (A) Ventricular (left) and auricular (right) areas of a *B*. *burgdorferi*–infected murine heart showing the relative abundance of distinct molecular markers. (B) PCA of the ventricular (green) and auricular (red) areas of an infected murine heart. (C) Representative ROCs and their corresponding auricular distribution (red dots) in *B*. *burgdorferi*–infected and uninfected (control) murine hearts. The top panels correspond to a marker of 5,311.92 Da, while the bottom panels represent a 7,017.2-Da marker. The data underlying the graphs in S1 Fig can be found in S1 Supporting Information. MALDI-IMS, MALDI Imaging; PCA, principal component analysis; ROC, receiving operating characteristic.
(TIF)

S2 Fig Comparison of regulated genes and proteins at 21 days of infection. The proteins regulated in the infected heart at 21 days of infection were matched with the transcriptional levels of their corresponding genes. (A) represents the differentially regulated genes that correspond to the differentially expressed proteins. (B) represents the regulated proteins. Genes and proteins labeled in blue correspond to those up-regulated, while those in red represent down-regulated genes/proteins. The data underlying the graphs in S2 Fig can be found in S8 Data, PXD019605, and GSE152168.
(TIF)

S3 Fig Transcriptomic profile of B. burgdorferi–stimulated murine macrophages. (A) Schematic representation of the working conditions to assess long-term effects of the stimulation of BMMs and human monocytes with *B*. *burgdorferi*. The 4 conditions were defined by the first and secondary stimulations, yielding the conditions UU, UB, BU, and BB. (B) Sample distance matrix of BMMs stimulated with *B*. *burgdorferi* under the conditions described in S2A Fig. (C) Volcano plot representing the DR of genes when unstimulated (UU) and stimulated and rested (BU) macrophages are compared. The red dots represent genes up-regulated (693), whereas the blue dots indicate down-regulated genes (641). (D) Venn diagram showing genes that are co- and differentially regulated in naïve (UB) and restimulated (BB) macrophages vs. unstimulated cells. The numbers at the top represent genes up-regulated, while those at the bottom indicate the number of genes down-regulated. (E) Venn diagrams including genes co- and differentially regulated among UB, BU, and BB macrophages when compared to unstimulated (UU) controls. The cutoff values to determine DE were set as an absolute value of log_2_ fold induction of 1 and Padj < 0.05. The data underlying the graphs in S3 Fig can be found in GSE125503. BMM, bone marrow-derived macrophage; DE, differential expression.
(TIF)

S4 Fig GFP presence in unstimulated and 48h stimulated BMMs. BMMs were incubated with GFP-containing *B*. *burgdorferi* for 48 hours or left untreated. The cells were washed and analyzed by flow cytometry for residual internalized GFP. The data presented are representative of 2–3 experiments. The data underlying the graphs in S4 Fig can be found in S8 Data. BMM, bone marrow-derived macrophage; GFP, green fluorescent protein.
(TIF)

S5 Fig Irf4-silenced RAW 264.7 cells regulate inflammatory output independently of phagocytosis. (A) Phagocytosis by RAW 264.7 cells containing sh*Irf4* (color histograms) compared to control, pLKO-infected cells (black histogram). The gray histogram represents the 4°C control. The cells were infected separately with 2 different shRNA sequences: TRCN0000081548 (48, red histogram, 52% silencing) and TRCN0000081549 (49; green histogram, 42% silencing). (B) TNF induction by *B*. *burgdorferi* stimulation for 16–20 hours in *Irf4*-silenced and control cells. *, *p* < 0.05. The data underlying the graphs in S5 Fig can be found in S8 Data. shRNA, short hairpin RNA; TNF, tumor necrosis factor.
(TIF)

S6 Fig 3-PO does not affect the growth of B. burgdorferi in vitro. Spirochetes (0.8 ×10^6^ per ml) were grown in the absence or presence of 0.1 and 1 μM of 3-PO for 5 days and counted. Control cultures contained equivalent amounts of DMSO (0.04 and 0.4%, respectively). The data underlying the graphs in S6 Fig can be found in S8 Data. 3-PO, 3-(3-pyridinyl)-1-(4-pyridinyl)-2-propen-1-one.
(TIF)

S7 Fig Variation in the number of macrophages upon their stimulation with B. burgdorferi. One million BMMs per well were plated in triplicate and stimulated following the schemed depicted in S3A Fig. The cells were counted after 48 hours of stimulation and at the end of the restimulation process. *, 1-way ANOVA, *p* < 0.05. The data underlying the graphs in S2 Fig can be found in S8 Data. ANOVA, analysis of variance; BMM, bone marrow-derived macrophage.
(TIF)

S1 Supporting Information MALDI-IMS data corresponding to the analysis of B. burgdorferi–infected and control, uninfected murine hearts. MALDI-IMS, MALDI Imaging. (ZIP)

S1 Data Excel file with the quantification data corresponding to Fig 1. (XLSX)

S2 Data Excel file with the quantification data corresponding to Fig 2. (XLSX)

S3 Data Excel file with the quantification data corresponding to Fig 3. (XLSX)

S4 Data Excel file with the quantification data corresponding to Fig 4. (XLSX)

S5 Data Excel file with the quantification data corresponding to Fig 5. (XLSX)

S6 Data Excel file with the quantification data corresponding to Fig 6. (XLSX)

S7 Data Excel file with the quantification data corresponding to Fig 7. (XLSX)

S8 Data Excel file with the quantification data corresponding to S2 and S4–S7 Figs. (XLSX)

S1 Table Enriched reactome pathways of proteins overrepresented in murine hearts infected with B. burgdorferi compared to uninfected controls. (DOCX)

S2 Table GAGE analysis of differentially regulated genes under the activated and rested (BU) and memory (BB) conditions. Only down-regulated pathways were found to be significantly different in activated and rested macrophages compared to memory cells. The statistic represents the measure of the cumulative fold-changes observed across samples within a given gene set. GAGE, Generally Applicable Gene-set Enrichment.
(DOCX)

S3 Table Genes overexpressed in memory macrophages compared to those acutely stimulated with B. burgdorferi that are putatively regulated by the transcription factors, SpiB, MAFK, and MZF1. (DOCX)

S4 Table Primers used. (DOCX)

S5 Table Upstream pathways regulated in experienced macrophages compared to acutely stimulated cells. Pathways activated (z-score >2) and repressed (z-score < −2) were identified using the IPA tool. IPA, Ingenuity Pathway Analysis.
(DOCX)

## Abbreviations

2-DG: 2-deoxyglucose
3-PO: 3-(3-pyridinyl)-1-(4-pyridinyl)-2-propen-1-one
ANOVA: analysis of variance
BCG: Bacille Calmette–Guérin
BMM: bone marrow-derived macrophage
CID: collision-induced dissociation
DE: differential expression
ECAR: extracellular acidification rate
FBS: fetal bovine serum
GAGE: Generally Applicable Gene-set Enrichment
GC–MS: gas chromatography–mass spectrometry
GFP: green fluorescent protein
GO-BP: Gene Ontology Biological Process
IL: interleukin
iNKT: invariant NKT
IPA: Ingenuity Pathway Analysis
LC–MS: liquid chromatography–mass spectrometry
MALDI-IMS: MALDI Imaging
MFI: mean fluorescence intensity
MOI: multiplicity of infection
MS: mass spectrometry
MS/MS: tandem mass spectrometry
NLR: nucleotide-binding oligomerization domain-like receptor
nt: nucleotide
OCR: oxygen consumption rate
OCT: optimal cutting temperature
PAMP: pathogen-associated molecular pattern
PCA: principal component analysis
PCR: polymerase chain reaction
PPAR: peroxisome proliferator-activated receptor
qPCR: quantitative polymerase chain reaction
RNA-seq: RNA sequencing
ROC: receiving operating characteristic
SE: standard error
shRNA: short hairpin RNA
TCA: tricarboxylic acid cycle
TLR: Toll-like receptor
TNF: tumor necrosis factor
ToF MS: time-of-flight mass spectrometer
