# Supplementary figures and images for: Borrelia burgdorferi infection induces long-term memory-like responses in macrophages with tissue-wide consequences in the heart
Source: PLoS Biol. 2021 Jan 4;19(1):e3001062. doi: 10.1371/journal.pbio.3001062 (PMC7808612; doi:10.1371/journal.pbio.3001062)

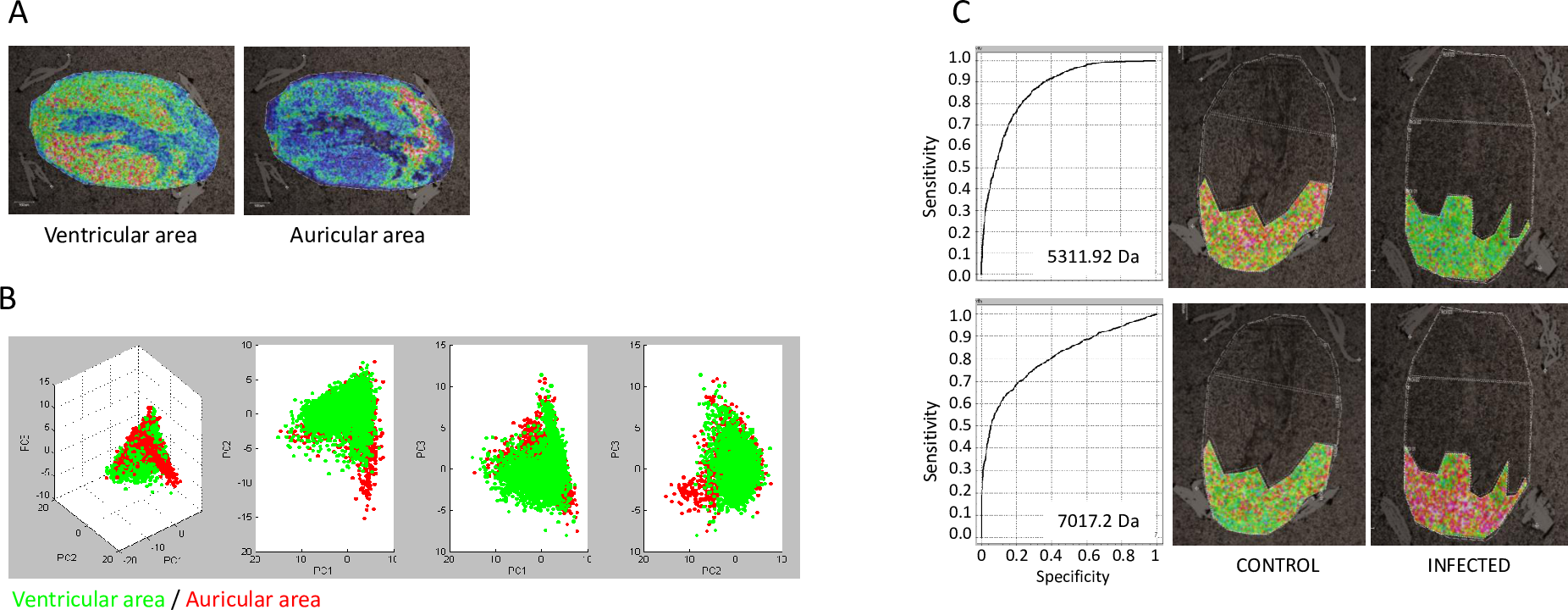

Supplement: S1 Fig — (A) Ventricular (left) and auricular (right) areas of a B. burgdorferi–infected murine heart showing the relative abundance of distinct molecular markers. (B) PCA of the ventricular (green) and auricular (red) areas of an infected murine heart. (C) Representative ROCs and their corresponding auricular distribution (red dots) in B. burgdorferi–infected and uninfected (control) murine hearts. The top panels correspond to a marker of 5,311.92 Da, while the bottom panels represent a 7,017.2-Da marker. The data underlying the graphs in S1 Fig can be found in S1 Supporting Information. MALDI-IMS, MALDI Imaging; PCA, principal component analysis; ROC, receiving operating characteristic. (TIF) [file pbio.3001062.s001.tif]

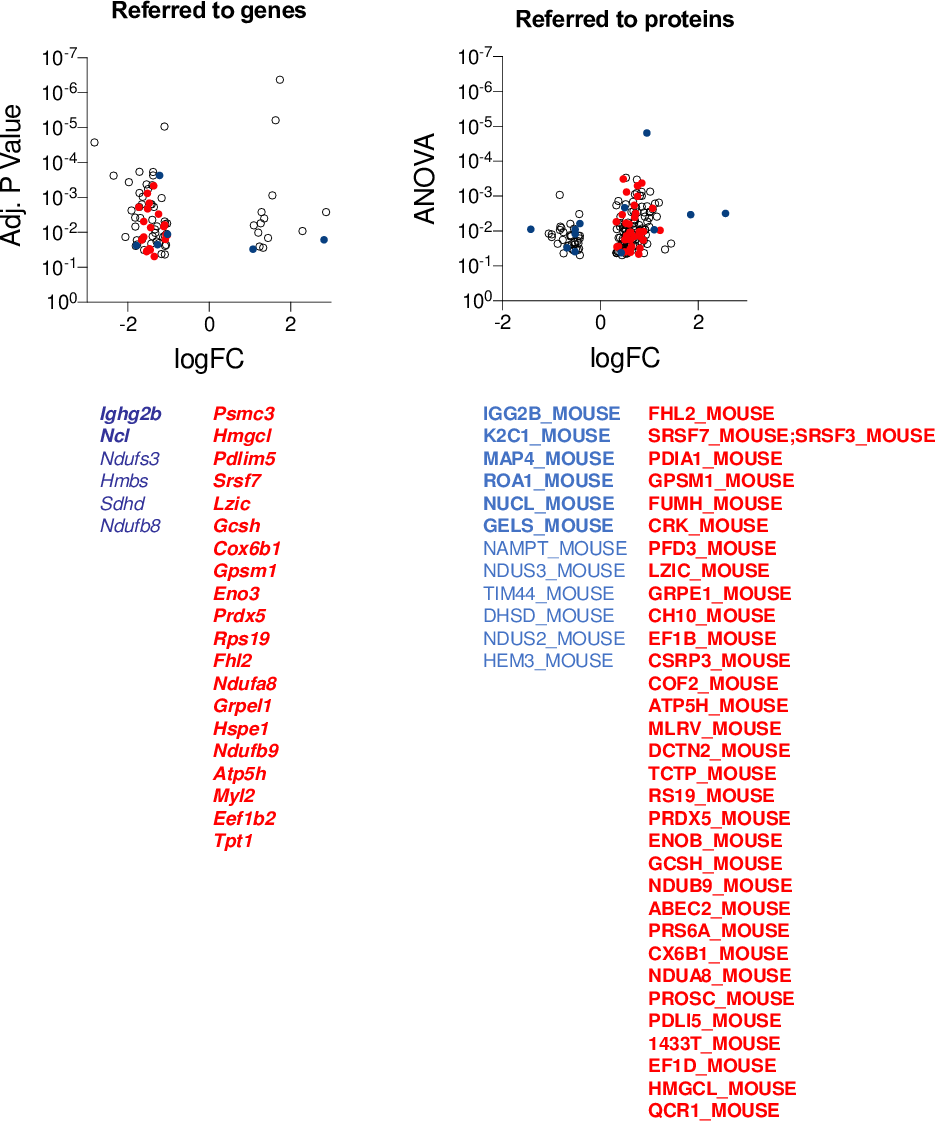

Supplement: S2 Fig — The proteins regulated in the infected heart at 21 days of infection were matched with the transcriptional levels of their corresponding genes. (A) represents the differentially regulated genes that correspond to the differentially expressed proteins. (B) represents the regulated proteins. Genes and proteins labeled in blue correspond to those up-regulated, while those in red represent down-regulated genes/proteins. The data underlying the graphs in S2 Fig can be found in S8 Data, PXD019605, and GSE152168. (TIF) [file pbio.3001062.s002.tif]

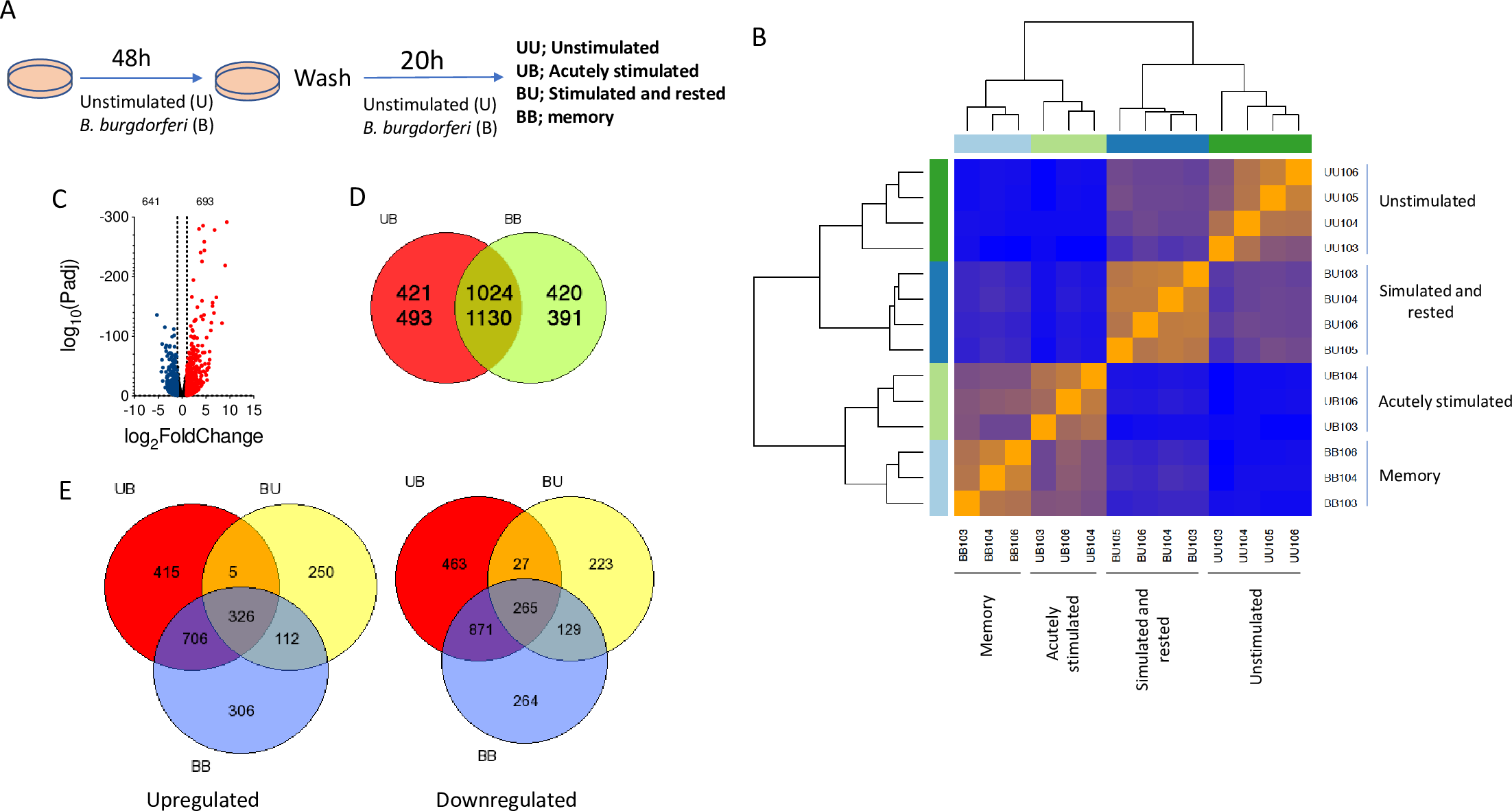

Supplement: S3 Fig — (A) Schematic representation of the working conditions to assess long-term effects of the stimulation of BMMs and human monocytes with B. burgdorferi. The 4 conditions were defined by the first and secondary stimulations, yielding the conditions UU, UB, BU, and BB. (B) Sample distance matrix of BMMs stimulated with B. burgdorferi under the conditions described in S2A Fig. (C) Volcano plot representing the DR of genes when unstimulated (UU) and stimulated and rested (BU) macrophages are compared. The red dots represent genes up-regulated (693), whereas the blue dots indicate down-regulated genes (641). (D) Venn diagram showing genes that are co- and differentially regulated in naïve (UB) and restimulated (BB) macrophages vs. unstimulated cells. The numbers at the top represent genes up-regulated, while those at the bottom indicate the number of genes down-regulated. (E) Venn diagrams including genes co- and differentially regulated among UB, BU, and BB macrophages when compared to unstimulated (UU) controls. The cutoff values to determine DE were set as an absolute value of log2 fold induction of 1 and Padj < 0.05. The data underlying the graphs in S3 Fig can be found in GSE125503. BMM, bone marrow-derived macrophage; DE, differential expression. (TIF) [file pbio.3001062.s003.tif]

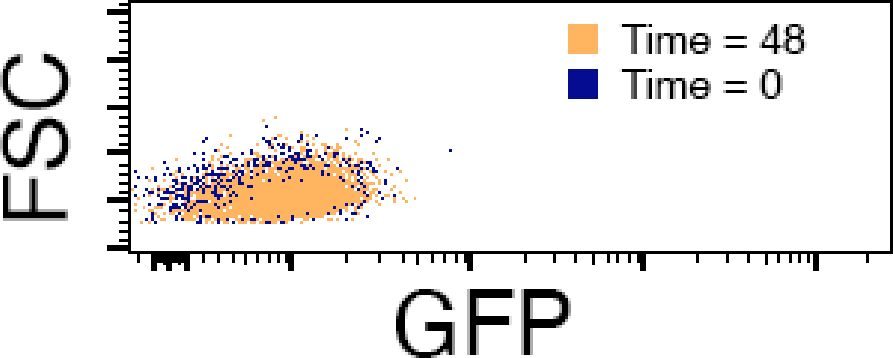

Supplement: S4 Fig — BMMs were incubated with GFP-containing B. burgdorferi for 48 hours or left untreated. The cells were washed and analyzed by flow cytometry for residual internalized GFP. The data presented are representative of 2–3 experiments. The data underlying the graphs in S4 Fig can be found in S8 Data. BMM, bone marrow-derived macrophage; GFP, green fluorescent protein. (TIF) [file pbio.3001062.s004.tif]

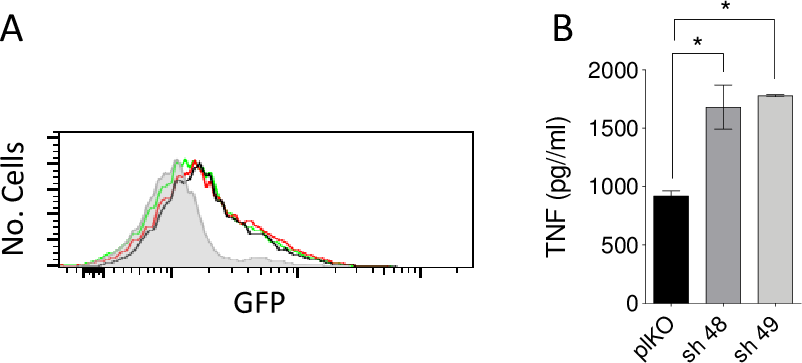

Supplement: S5 Fig — (A) Phagocytosis by RAW 264.7 cells containing shIrf4 (color histograms) compared to control, pLKO-infected cells (black histogram). The gray histogram represents the 4°C control. The cells were infected separately with 2 different shRNA sequences: TRCN0000081548 (48, red histogram, 52% silencing) and TRCN0000081549 (49; green histogram, 42% silencing). (B) TNF induction by B. burgdorferi stimulation for 16–20 hours in Irf4-silenced and control cells. *, p < 0.05. The data underlying the graphs in S5 Fig can be found in S8 Data. shRNA, short hairpin RNA; TNF, tumor necrosis factor. (TIF) [file pbio.3001062.s005.tif]

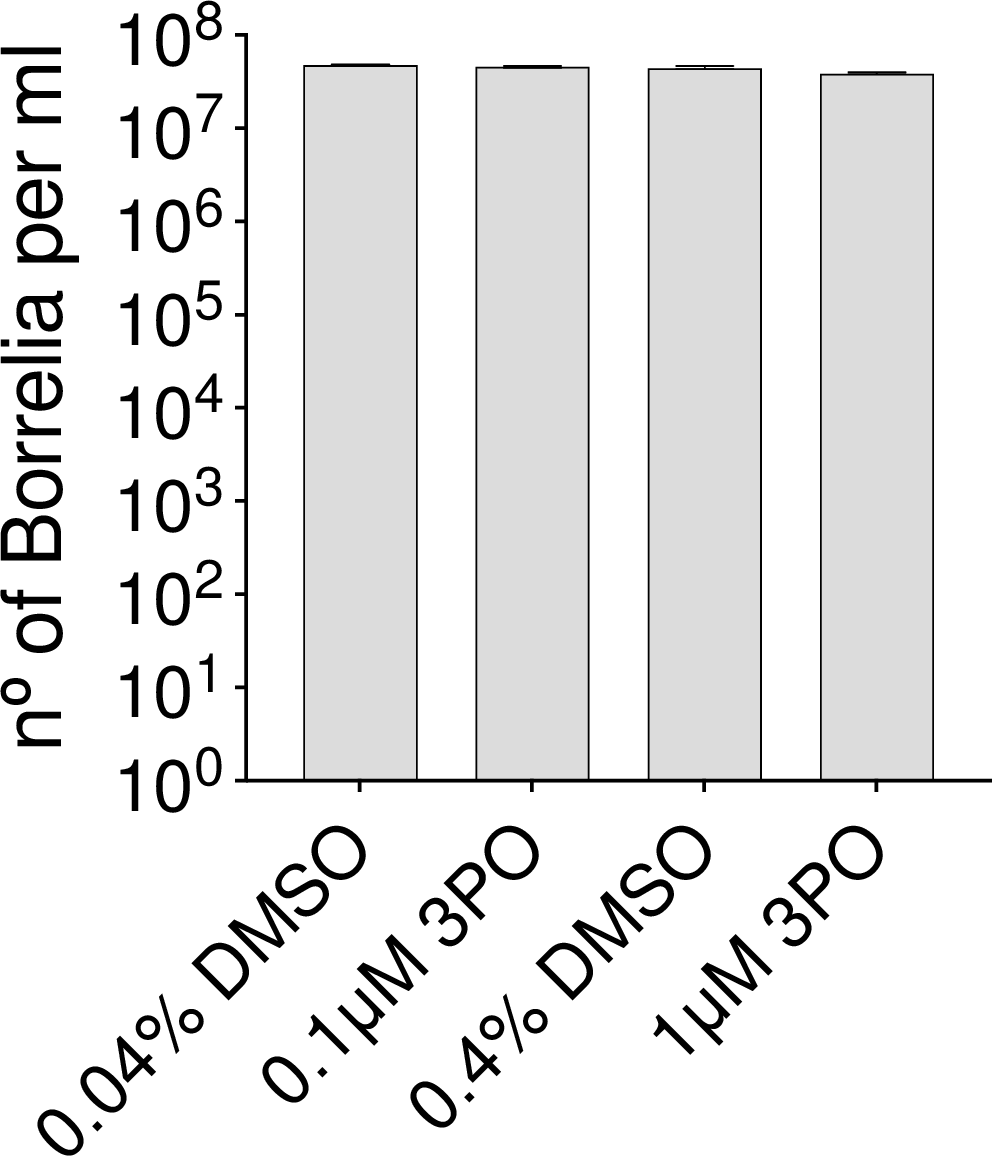

Supplement: S6 Fig — Spirochetes (0.8 ×10^6^ per ml) were grown in the absence or presence of 0.1 and 1 μM of 3-PO for 5 days and counted. Control cultures contained equivalent amounts of DMSO (0.04 and 0.4%, respectively). The data underlying the graphs in S6 Fig can be found in S8 Data. 3-PO, 3-(3-pyridinyl)-1-(4-pyridinyl)-2-propen-1-one. (TIF) [file pbio.3001062.s006.tif]

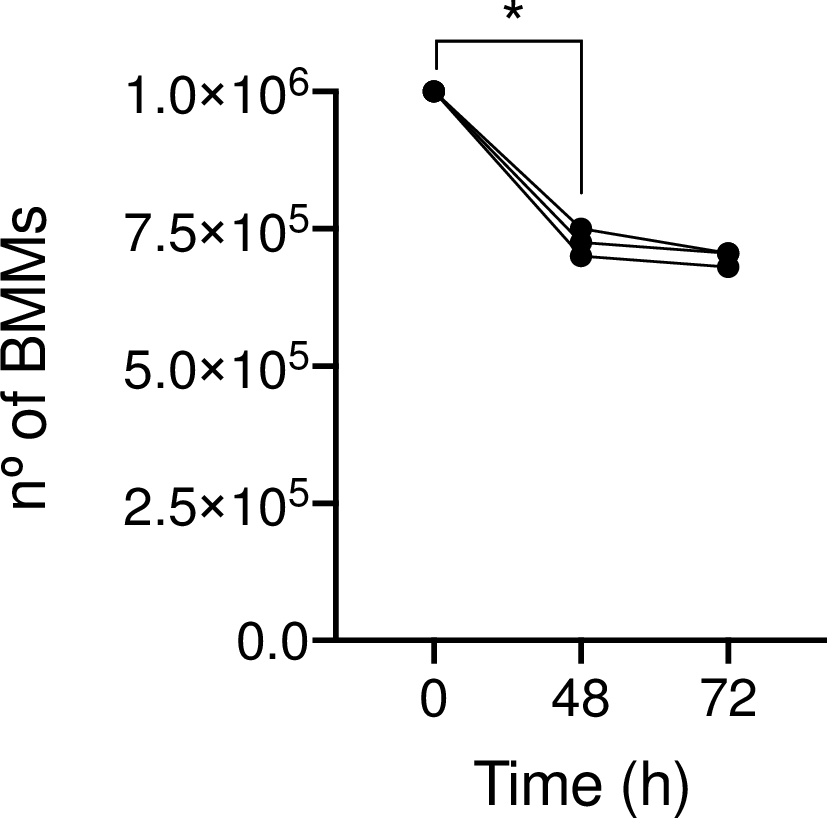

Supplement: S7 Fig — One million BMMs per well were plated in triplicate and stimulated following the schemed depicted in S3A Fig. The cells were counted after 48 hours of stimulation and at the end of the restimulation process. *, 1-way ANOVA, p < 0.05. The data underlying the graphs in S2 Fig can be found in S8 Data. ANOVA, analysis of variance; BMM, bone marrow-derived macrophage. (TIF) [file pbio.3001062.s007.tif]
