# Supplementary material for: Borrelia burgdorferi infection induces long-term memory-like responses in macrophages with tissue-wide consequences in the heart
Source: PLoS Biol. 2021 Jan 4;19(1):e3001062. doi: 10.1371/journal.pbio.3001062 (PMC7808612; doi:10.1371/journal.pbio.3001062)
Supplement: S1 Table — (DOCX) [file pbio.3001062.s017.docx]

**Table S1. Enriched Reactome pathways of proteins over represented in murine hearts infected with *B. burgdorferi* compared to uninfected controls.**

| **Term ID** | | **Term Description** | | **Observed Gene Count** | | **Background Gene Count** | | **FDR** | |
| --- | --- | --- | --- | --- | --- | --- | --- | --- | --- |
| MMU-390522 | Striated Muscle Contraction | | 9 | | 31 | | 1.59e-09 | |  |
| MMU-1428517 | The citric acid (TCA) cycle and respiratory electron transport | | 13 | | 129 | | 2.04e-09 | |  |
| MMU-163200 | Respiratory electron transport, ATP synthesis by chemiosmotic coupling, and heat production by uncoupling proteins. | | 10 | | 78 | | 4.16e-08 | |  |
| MMU-397014 | Muscle contraction | | 11 | | 162 | | 1.99e-06 | |  |
| MMU-611105 | Respiratory electron transport | | 7 | | 56 | | 1.44e-05 | |  |
