# Supplementary material for: Borrelia burgdorferi infection induces long-term memory-like responses in macrophages with tissue-wide consequences in the heart
Source: PLoS Biol. 2021 Jan 4;19(1):e3001062. doi: 10.1371/journal.pbio.3001062 (PMC7808612; doi:10.1371/journal.pbio.3001062)
Supplement: S2 Table — Only down-regulated pathways were found to be significantly different in activated and rested macrophages compared to memory cells. The statistic represents the measure of the cumulative fold-changes observed across samples within a given gene set. GAGE, Generally Applicable Gene-set Enrichment. (DOCX) [file pbio.3001062.s018.docx]

**Table S2. Generally applicable gene enrichment (GAGE) analysis of differentially regulated genes under the activated and rested (BU) and memory (BB) conditions.** Only downregulated pathways were found to be significantly different in activated and rested macrophages compared to memory cells. The statistic represents the measure of the cumulative fold-changes observed across samples within a given gene set.

| **Direction** | **GAGE analysis: BU vs BB** | **Statistic** | **Nº of genes** | **adj. Pval** |
| --- | --- | --- | --- | --- |
| Down | Response to bacterium | -5.1763 | 418 | 4.3e-04 |
|  | Regulation of defense response | -5.1054 | 457 | 4.3e-04 |
|  | Innate immune response | -4.7785 | 493 | 1.3e-03 |
|  | Positive regulation of defense response | -4.772 | 281 | 1.3e-03 |
|  | Response to molecule of bacterial origin | -4.7147 | 263 | 1.3e-03 |
|  | Response to lipopolysaccharide | -4.6514 | 251 | 1.3e-03 |
|  | Inflammatory response | -4.6417 | 437 | 1.3e-03 |
|  | Defense response to other organism | -4.6053 | 307 | 1.3e-03 |
|  | Regulation of signaling receptor activity | -4.3582 | 216 | 4.1e-03 |
|  | Cytokine-mediated signaling pathway | -4.2859 | 268 | 4.5e-03 |
