# Supplementary material for: Borrelia burgdorferi infection induces long-term memory-like responses in macrophages with tissue-wide consequences in the heart
Source: PLoS Biol. 2021 Jan 4;19(1):e3001062. doi: 10.1371/journal.pbio.3001062 (PMC7808612; doi:10.1371/journal.pbio.3001062)
Supplement: S3 Table — (DOCX) [file pbio.3001062.s019.docx]

Table S3. Genes overexpressed in memory macrophages compared to those acutely stimulated with *B. burgdorferi* that are putatively regulated by the transcription factors, SpiB, MAFK and MZF1.

| **SpiB** | | | **MAFK** | **MZF1** | |
| --- | --- | --- | --- | --- | --- |
| *Adam8* | *Fcrl5* | *Pald1* | *9930111J21Rik2* | *Arg1* | *Rapgef5* |
| *Aldoc* | *Fgd2* | *Phlda3* | *Ccr7* | *Arhgap4* | *Shroom4* |
| *Atp2b4* | *Gapt* | *Pld4* | *Gm19705* | *Cd207* | *Siglece* |
| *BC147527* | *Gatsl2* | *Rasgrp4* | *Gm5431* | *Dab2* | *Spata13* |
| *Ccl7* | *Gbgt1* | *Sirpb1b* | *Ifi202b* | *Dusp2* | *Tiam1* |
| *Cd80* | *Gm5615* | *Slc9a9* | ***Irf4*** | *Fxyd2* | *Tob1* |
| *Cd86* | *Gm9733* | *Tlr9* | *Klra2* | *Gm9992* | *Trem1* |
| *Cfp* | *Gpr31b* | *Vegfa* | *Pxk* | *Havcr2* |  |
| *Clec4a1* | *Gpr35* |  | *S100a8* | *Hhex* |  |
| *Clec4a2* | *Havcr2* |  | *Sall2* | ***Irf4*** |  |
| *Clec4b1* | *Ifitm6* |  | *Sh2d2a* | *Itga6* |  |
| *Dock2* | *Inpp4b* |  | *Smad1* | *Lgmn* |  |
| *Dpy19l3* | *Iqsec3* |  | *Tnfrsf22* | *Lpar6* |  |
| *Dpysl2* | ***Irf4*** |  | *Tnfrsf23* | *Pgm2l1* |  |
| *F11r* | *Kctd12* |  |  |  |  |
| *Fam129a* | *Klra2* |  |  |  |  |
| *Fam43a* | *Lilra5* |  |  |  |  |
| *Fcgr1* | *Lrrc4* |  |  |  |  |
