# Supplementary material for: Borrelia burgdorferi infection induces long-term memory-like responses in macrophages with tissue-wide consequences in the heart
Source: PLoS Biol. 2021 Jan 4;19(1):e3001062. doi: 10.1371/journal.pbio.3001062 (PMC7808612; doi:10.1371/journal.pbio.3001062)
Supplement: S4 Table — (DOCX) [file pbio.3001062.s020.docx]

Table S4. Primers used.

| **Gene** | **Primers** | | | **Source** | |
| --- | --- | --- | --- | --- | --- |
| Mouse: | |  |  |  |  |
| *Gapdh* | 5’-TTGATGGCAACAATCTCCAC-3’  5’-CGTCCCGTAGACAAAATGG-3’ | | | [1] | |
| *Rpl19* | 5’-GACCAAGGAAGCACGAAAGC-3’  5’-CAGGCCGCTATGTACAGACA-3’ | | | [2] | |
| *Marco* | 5’-TTAGCAGCTATGGAGGTGGC-3’  5’-GACACACTGATGACCTCTCGG-3’ | | | [2] | |
| *Irf4* | 5’-GAGTAGGATCTACTGGGATG-3’  5’-CTTGCAGCTCTGATAGAAAC-3’ | | | Sigma-Aldrich | |
| *Adgre1* | 5’-CTGCGCAGATGTTGATGAGTGTC-3’  5’-GGAGCCATTCAAGACAAAGCCTG-3’ | | | [3] | |
| *Tnf* | 5’-AGCCCACGTCGTAGCAAACCAC-3’  5’-ATCGGCTGGCACCACTAGTTGGT-3’ | | | [3] | |
| Human: | | | | |  |
| *LDHA* | 5’-CACCATGATTAAGGGTCTTTAC-3’  5’-AGGTCTGAGATTCCATTCTG-3’ | | | Sigma-Aldrich | |
| *B. burgdorferi*: | | | | |  |
| *recA* | 5’-GTGGATCTATTGTATTAGATGAGGCT-3’  5’-GCCAAAGTTCTGCAACATTAACACCT-3’ | | | [4] | |

1. Barbier-Torres L, Fortner KA, Iruzubieta P, Delgado TC, Giddings E, Chen Y, et al. Silencing hepatic MCJ attenuates non-alcoholic fatty liver disease (NAFLD) by increasing mitochondrial fatty acid oxidation. Nat Commun. 2020;11:3360.

2. Carreras-González A, Barriales D, Palacios A, Montesinos-Robledo M, Navasa N, Azkargorta M, et al. Regulation of macrophage activity by surface receptors contained within Borrelia burgdorferi-enriched phagosomal fractions. PLoS Pathogens. 2019;In press.

3. Carreras-Gonzalez A, Navasa N, Martin-Ruiz I, Lavin JL, Azkargorta M, Atondo E, et al. A multi-omic analysis reveals the regulatory role of CD180 during the response of macrophages to Borrelia burgdorferi. Emerg Microbes Infect. 2018;7:19.

4. Morrison TB, Ma Y, Weis JH, Weis JJ. Rapid and sensitive quantification of *Borrelia burgdorferi*-infected mouse tissues by continuous fluorescent monitoring of PCR. J Clin Microbiol. 1999;37:987-92.
