# Supplementary material for: Borrelia burgdorferi infection induces long-term memory-like responses in macrophages with tissue-wide consequences in the heart
Source: PLoS Biol. 2021 Jan 4;19(1):e3001062. doi: 10.1371/journal.pbio.3001062 (PMC7808612; doi:10.1371/journal.pbio.3001062)
Supplement: S5 Table — Pathways activated (z-score >2) and repressed (z-score < −2) were identified using the IPA tool. IPA, Ingenuity Pathway Analysis. (DOCX) [file pbio.3001062.s021.docx]

Table S5. Upstream pathways regulated in experienced macrophages compared to acutely stimulated cells. Pathways activated (z-score >2) and repressed (z-score < -2) were identified using the Ingenuity Pathway Analysis tool.

| **Upstream Regulator** | **Expr Log Ratio** | **Activation z-score** | **p-value of overlap** |
| --- | --- | --- | --- |
| Prostaglandin E2 |  | 3.843 | 5.92E-29 |
| *Il6* | 1.569 | 3.7 | 5.67E-35 |
| Cg |  | 3.294 | 4.86E-14 |
| ***Hif1a*** | 0.685 | 3.223 | 2.74E-15 |
| *Stat3* | 0.479 | 3.165 | 1.63E-25 |
| Ca^2+^ |  | 3.156 | 1.1E-10 |
| FGF2 |  | 3.084 | 1.98E-15 |
| Forskolin |  | 3.045 | 4.73E-19 |
| Mek |  | 3.04 | 2.76E-09 |
| *Cd38* | 1.002 | 3.003 | 7.84E-07 |
| *Mgea5* | -0.289 | 2.991 | 2.52E-13 |
| *Ptger4* | 0.68 | 2.954 | 4.06E-21 |
| PDGF BB |  | 2.917 | 2.24E-17 |
| *Il5* | -0.233 | 2.887 | 1.44E-17 |
| Haloperidol |  | 2.884 | 9.73E-05 |
| C1q |  | 2.813 | 4.66E-09 |
| Diethylstilbestrol |  | 2.774 | 3.15E-08 |
| PI3K (complex) |  | 2.774 | 9.44E-16 |
| Creb |  | 2.769 | 2.21E-09 |
| Lipopolysaccharide |  | 2.759 | 7.87E-57 |
| Lh |  | 2.748 | 6.12E-04 |
| *Cebpe* | 0.025 | 2.715 | 1.93E-10 |
| *Il1b* | -0.629 | 2.663 | 1.44E-36 |
| Dalfampridine |  | 2.646 | 1.50E-04 |
| *Il2* | -0.043 | 2.642 | 1.65E-29 |
| *Csf3* | 0.595 | 2.634 | 3.96E-21 |
| *Pou2f2* | 0.415 | 2.598 | 1.21E-03 |
| Pdgf (complex) |  | 2.554 | 2.21E-13 |
| Bee venom |  | 2.53 | 2.84E-10 |
| *Map3k8* | 0.18 | 2.476 | 3.22E-15 |
| CALCA |  | 2.468 | 2.56E-08 |
| PI3K (family) |  | 2.465 | 1.43E-10 |
| *Tgfb1* | -0.511 | 2.463 | 2.63E-30 |
| Inosine |  | 2.4 | 4.00E-03 |
| Salmeterol |  | 2.376 | 3.44E-06 |
| Amphetamine |  | 2.375 | 3.38E-04 |
| Phorbol esters |  | 2.372 | 7.27E-05 |
| *Areg* | 0.101 | 2.356 | 1.12E-03 |
| MYCN |  | 2.356 | 1.95E-07 |
| Histamine |  | 2.327 | 1.98E-10 |
| TSH |  | 2.321 | 9.60E-04 |
| Doxorubicin |  | 2.283 | 1.13E-04 |
| *Igf1* | 0.385 | 2.248 | 6.95E-16 |
| 2,4-dinitrophenol-ovalbumin conjugate |  | 2.236 | 2.04E-03 |
| *Tgfbr2* | 0.685 | 2.228 | 1.44E-09 |
| IL4 |  | 2.225 | 2.68E-40 |
| Lipoteichoic acid |  | 2.225 | 1.56E-11 |
| *Kat5* | -0.298 | 2.219 | 1.14E-02 |
| VitaminD3-VDR-RXR |  | 2.219 | 9.99E-03 |
| Methotrexate |  | 2.21 | 4.16E-14 |
| 6-hydroxydopamine |  | 2.203 | 4.60E-06 |
| *Gdnf* | -0.31 | 2.199 | 4.68E-06 |
| *Ins1* | 0.465 | 2.19 | 4.31E-09 |
| *Lif* | -0.362 | 2.185 | 3.09E-07 |
| Bicuculline |  | 2.183 | 9.50E-5 |
| *Prkaca* | 0.091 | 2.183 | 2.16E-03 |
| Beta-estradiol |  | 2.182 | 1.84E-25 |
| *Il6st* | -0.495 | 2.177 | 1.78E-03 |
| *F2r* | 0.165 | 2.176 | 5.67E-10 |
| bucladesine |  | 2.173 | 1.08E-06 |
| *Nfil3* | 0.714 | 2.169 | 1.29E-03 |
| TGFA |  | 2.168 | 2.13E-10 |
| *Klf5* | 0.048 | 2.163 | 4.82E-03 |
| *Fgf4* | -0.348 | 2.155 | 4.22E-04 |
| *Egfr* | 0.305 | 2.152 | 9.73E-07 |
| *Erg* | 0.025 | 2.138 | 1.43E-05 |
| Formaldehyde |  | 2.138 | 1.76E-03 |
| Atorvastatin |  | 2.136 | 1.14E-09 |
| *Ptpn11* | 0.212 | 2.131 | 1.62E-02 |
| Epinephrine |  | 2.13 | 3.50E-05 |
| *Myc* | 0.323 | 2.124 | 2.66E-14 |
| ATP |  | 2.121 | 5.84E-15 |
| *Nedd9* | -0.039 | 2.121 | 1.24E-05 |
| *Foxm1* | -0.234 | 2.107 | 5.46E-05 |
| 17-alpha-ethinylestradiol |  | 2.104 | 4.28E-11 |
| phorbol myristate acetate |  | 2.102 | 7.95E-25 |
| ERK |  | 2.101 | 2.00E-14 |
| *Sst* | -1.034 | 2.098 | 6.07E-05 |
| GnRH-A |  | 2.071 | 4.89E-05 |
| *Tgfb2* | -0.567 | 2.069 | 7.82E-09 |
| PTHLH |  | 2.062 | 2.33E-07 |
| *Eif4e* | -0.099 | 2.055 | 4.50E-05 |
| *Pdgfb* | -0.609 | 2.051 | 5.88E-04 |
| Deferoxamine |  | 2.04 | 3.47E-13 |
| INS | -0.214 | 2.018 | 3.34E-05 |
| Nicotine |  | 2.008 | 4.07E-11 |
| Collagen type IV |  | 2 | 3.27E-03 |
| Hydroxyurea |  | 2 | 1.07E-01 |
| *Kdm8* | 0.078 | 2 | 1.23E-04 |
| Methylnitronitrosoguanidine |  | 2 | 1.74E-02 |
| *Muc1* | 0.467 | 2 | 3.30E-06 |
| p38 Sapk |  | 2 | 3.30E-06 |
| Poly-L-lysine |  | 2 | 3.92E-04 |
| Ponesimod |  | 2 | 4.31E-05 |
| 1L-6-hydroxymethyl-chiro-inositol 2-(R)-2-O-methyl-3-O-octadecylcarbonate |  | -2 | 7.57E-05 |
| *Acvrl1* | 0.048 | -2 | 5.13E-04 |
| *Atp2a2* | -0.13 | -2 | 2.73E-03 |
| Brodalumab |  | -2 | 5.37E-04 |
| Diazoxide |  | -2 | 2.69E-03 |
| *Mkl2* | -0.326 | -2 | 1.12E-02 |
| *Peli1* | -0.168 | -2.018 | 4.30E-06 |
| *Sirt3* | 0.135 | -2.024 | 4.13E-04 |
| Curcumin |  | -2.045 | 4.74E-15 |
| SB-431542 |  | -2.111 | 1.17E-04 |
| Lisinopril |  | -2.121 | 1.49E-07 |
| Losartan potassium |  | -2.14 | 4.60E-05 |
| *Cx3cl1* | -0.238 | -2.177 | 9.70E-05 |
| *Vhl* | 0.231 | -2.181 | 1.44E-09 |
| Fulvestrant |  | -2.193 | 6.03E-11 |
| KLRC4-KLRK1/KLRK1 |  | -2.197 | 8.94E-05 |
| *Ciita* | -1.717 | -2.204 | 5.97E-04 |
| *Snai1* | 0.313 | -2.207 | 4.45E-02 |
| Pde4 |  | -2.208 | 1.60E-04 |
| KN 93 |  | -2.213 | 1.29E-03 |
| *Glrx* | 1.588 | -2.219 | 1.00E-6 |
| *Ptprc* | -0.186 | -2.219 | 2.96E-06 |
| W7 |  | -2.236 | 2.06E-05 |
| Diclofenac |  | -2.242 | 8.67E-08 |
| Emodin |  | -2.243 | 2.17E-10 |
| *Ptpn1* | -0.558 | -2.309 | 7.59E-08 |
| LY294002 |  | -2.482 | 1.15E-18 |
| *Ccr5* | 0.358 | -2.505 | 6.76E-11 |
| *Pten* | 0.266 | -2.572 | 2.56E-12 |
| Beta-naphthoflavone |  | -2.596 | 7.64E-04 |
| Androgen |  | -2.646 | 3.86E-05 |
| NS-398 |  | -2.673 | 1.11E-7 |
| Silibinin |  | -2.742 | 1.26E-03 |
| CD3 |  | -2.811 | 3.3E-15 |
| Salirasib |  | -2.813 | 1.26E-04 |
| *Stk11* | 0.089 | -2.88 | 9.04E-04 |
| AP5 |  | -2.882 | 2.09E-05 |
| EGTA |  | -2.961 | 2.91E-07 |
| Linsidomine |  | -2.961 | 5.92E-07 |
| H89 |  | -3.285 | 1,01E-13 |
